# The Incidence of *Wolbachia* Bacterial Endosymbiont in Bisexual and Parthenogenetic Populations of the Psyllid Genus *Cacopsylla* (Hemiptera, Psylloidea)

**DOI:** 10.3390/insects12100853

**Published:** 2021-09-22

**Authors:** Nazar A. Shapoval, Seppo Nokkala, Christina Nokkala, Galina N. Kuftina, Valentina G. Kuznetsova

**Affiliations:** 1 Department of Karyosystematics, Zoological Institute, Russian Academy of Sciences, Universitetskaya nab. 1, 199034 Saint-Petersburg, Russia; galinakuftina@mail.ru; 2 Laboratory of Genetics, Department of Biology, University of Turku, FI-20014 Turku, Finland; sepnok@utu.fi (S.N.); chrinok@utu.fi (C.N.)

**Keywords:** *Wolbachia* infection, phylogeography, infection frequency, PCR screening, bisexual reproduction, parthenogenesis, jumping plant-lice

## Abstract

**Simple Summary:**

*Wolbachia* has many varied impacts on the biology and evolution of insects and some other groups of invertebrates. The number of studies that have particularly addressed the impact of *Wolbachia* infection on reproduction and processes of speciation and diversification of host species has grown rapidly over the past decade. Despite that, our current knowledge on *Wolbachia* is limited, and comprehensive large-scale biogeographical studies devoted to the incidence of *Wolbachia* within a certain taxon or groups of taxa, although they are of great importance, are still scarce. In the present study, we focused on several Palaearctic *Cacopsylla* (Hemiptera, Psylloidea) species with different (parthenogenetic and bisexual) reproductive strategies. We conducted PCR screening of 526 specimens collected in different geographical regions of Europe and Russia in order to estimate a broad pattern of *Wolbachia* incidence and prevalence of five *Cacopsylla* species, *Cacopsylla. borealis*, *Cacopsylla. lapponica*, *Cacopsylla. myrtilli*, *Cacopsylla. ledi*, and *Cacopsylla. fraudatrix*. We revealed significant differences in infection frequencies between the species and even distinct populations of the same species, which, however, did not correlate with reproduction strategy or gender. These findings provide a starting point for understanding the role of *Wolbachia* infection in Cacopsylla evolution and diversity.

**Abstract:**

*Wolbachia* is one of the most common intracellular bacteria; it infects a wide variety of insects, other arthropods, and some nematodes. *Wolbachia* is ordinarily transmitted vertically from mother to offspring and can manipulate physiology and reproduction of their hosts in different ways, e.g., induce feminization, male killing, and parthenogenesis. Despite the great interest in *Wolbachia*, many aspects of its biology remain unclear and its incidence across many insect orders, including Hemiptera, is still poorly understood. In this report, we present data on *Wolbachia* infection in five jumping plant-lice species (Hemiptera, Psylloidea) of the genus *Cacopsylla* Ossiannilsson, 1970 with different reproductive strategies and test the hypothesis that *Wolbachia* mediates parthenogenetic and bisexual patterns observed in some *Cacopsylla* species. We show that the five species studied are infected with a single *Wolbachia* strain, belonging to the supergroup B. This strain has also been found in different insect orders (Lepidoptera, Hemiptera, Plecoptera, Orthoptera, Hymenoptera, Diptera) and even in acariform mites (Trombidiformes), suggesting extensive horizontal transmission of *Wolbachia* between representatives of these taxa. Our survey did not reveal significant differences in infection frequency between parthenogenetic and bisexual populations or between males and females within bisexual populations. However, infection rate varied notably in different *Cacopsylla* species or within distinct populations of the same species. Overall, we demonstrate that *Wolbachia* infects a high proportion of *Cacopsylla* individuals and populations, suggesting the essential role of this bacterium in their biology.

## 1. Introduction

*Wolbachia* Hertig 1936 is a diverse genus of intracellular rickettsia bacteria (Alphaproteobacteria: Rickettsiales: Rickettsiaceae) that infects invertebrates, namely arthropods and nematodes [1]. Representatives of the genus were found for the first time in 1924 by M. Hertig and S. Wolbach [2] in the mosquito *Culex pipiens* Linnaeus, 1758. Subsequently, the type species for the *Wolbachia* genus, *W. pipientis* Hertig, 1936, was formally described [3]. *Wolbachia* is considered one of the most common and widespread endosymbiotic bacterium so far discovered, infecting a broad range of hosts. Estimates suggest that more than 65% of all insect species are infected with *Wolbachia* and these bacteria are also characteristic of other arthropods, such as arachnids and crustaceans, and of nematodes [4,5,6]. *Wolbachia* is genetically diverse and, as of now, is generally divided into 16–17 main monophyletic lineages = “supergroups” (A to S) [7]. Members of some supergroups are exclusively found in filarial (C, D, J) or plant (L) parasitic nematodes [8,9,10,11,12], whereas supergroup F consists of strains infecting both nematodes and arthropods [13,14]. Members of all other supergroups (clades G and R are now considered to be part of the supergroups B and A, respectively [15]) infect only arthropods [16,17,18,19,20].

*Wolbachia* is mainly transmitted vertically from mother to offspring, while its lateral or horizontal transmission between host species has also been recorded [5,21,22,23,24]. *Wolbachia* strains associated with nematodes generally evolve mutualistic interactions with their hosts. In some filarial nematodes, *Wolbachia* bacteria are beneficial to the host and treated as primary (obligate) symbionts that have coevolved from ancient associations; they show within and among species prevalence and co-phylogeny with the host. In these associations, *Wolbachia* seems to be exclusively vertically transmitted, which is regarded as a sign of host-provided benefits [25]. *Wolbachia* members involved in relationships with insects and other arthropods may have no evident impact on their hosts; however, in some cases they are able to distort host sex ratios toward females. Their prevalence varies within and among taxa, showing no co-speciation events. The only proven exception to this rule is the common bedbug *Cimex lectularius* Linnaeus, 1758 (Hemiptera). This species, and probably its congeners, have demonstrated beneficial relationships with *Wolbachia*, which are based on mutualistic nutritional provision and co-cladogenesis with it [26].

*Wolbachia* endosymbionts are usually present in the reproductive tissues of the host species. Their effects include ***feminization*** of genetic males, that develop as females (this phenomenon is common in isopod crustaceans and in insect orders Lepidoptera and Hemiptera); ***male killing***—elimination of male progeny to improve the surviving advantage of infected female siblings (insect orders Diptera, Coleoptera, and Lepidoptera, and also arachnid order Pseudoscorpiones); ***sperm–egg cytoplasmic incompatibility***—unsuccessful mating of infected males with uninfected females or females that harbor different *Wolbachia* strains (Acari, Isopoda, and insect orders Coleoptera, Diptera, Hemiptera, Hymenoptera, Lepidoptera, and Orthoptera); ***parthenogenetic induction*** (=*Wolbachia*-induced thelytokous parthenogenesis)—development of unfertilized eggs and elimination of males from reproduction (Acari, Hymenoptera, Thysanoptera). In addition to the well-known effects listed above, infection with *Wolbachia* is known to cause changes at the cytological level, such as defects in chromosome condensation and segregation, disfunction of the centrosome, delays in mitotic progression, and nuclear envelope breakdown [27,28]. In some cases, infection provides a fitness benefit to the host. As observations of *Drosophila melanogaster* Meigen, 1830 suggest, infected females show an increased frequency of meiotic recombination and higher reproductive output than *Wolbachia*-free flies [29]. Recent reports on isopod species suggested that *Wolbachia*-to-host horizontal genome transfer may cause the evolutionary transitions in sex chromosome systems in animals, including the emergence of sex chromosomes *de novo* [24,30].

*Wolbachia* is characterized by relatively small genome size (1.08–1.7 Mb). Such reduction of the genome is typical for the Rickettsiales and most likely developed as a host adaptation. Assembling of *Wolbachia* genomes revealed that parasitic strains may contain a high number of repetitive and mobile elements [31]. In contrast, mutualistic filarial nematode *Wolbachia* lacks repetitive elements, or their number is significantly reduced [32]. Another remarkable feature is that *Wolbachia* possesses a high level of nucleotide divergence, in certain genes (e.g., *wsp* gene) exceeding 43%. Such remarkable genetic diversity could be explained by the extensive recombination between distinct strains. Both features, a large amount of repetitive DNA and high recombination rates of various regions of the genome, may be actively selected as a mechanism that facilitates successful establishment of parasitic *Wolbachia* within novel host lineages after horizontal transmission. Some of these highly divergent genes (e.g., *wsp* gene coding *Wolbachia* surface proteins and its two paralogues, *wspA* and *wspB*) together with *16S rRNA* and *ftsZ* genes traditionally serve as molecular markers to detect *Wolbachia* infection [33].

Jumping plant-lice or psyllids (Psylloidea) are classified as a superfamily of Sternorrhyncha (Hemiptera), comprising over 3800 described species of small plant-sap feeding insects [34]. Several psyllid species, such as the Asian citrus psyllid *Diaphorina citri* Kuwayama, 1908 (Liviidae), *Mycopsylla* spp. (Homotomidae), and *Psyllopsis* spp. (Psyllidae) have been shown to be infected with *Wolbachia* [35,36]. For example, all 15 sampled populations of *D. citri* in Brazil were completely infected with *Wolbachia* [35].

*Cacopsylla* Ossiannilsson, 1970 is one of the largest psyllid genera, with approximately 170 described taxa associated with woody dicotyledonous plants, consistent with the majority of psyllids in general. The genus has a predominantly Holarctic distribution with a few species occurring in the Afrotropical, Oriental, and Australasian biogeographical regions [37,38,39]. A series of *Cacopsylla* species possesses a kind of reproductive strategy where, in some populations, males and females are equally abundant, in other populations males are exclusively rare, and in some further populations only females are found [40,41,42,43,44,45,46,47,48]. The genus *Cacopsylla* offers, thus, an attractive model system to investigate the co-existence of different reproductive modes within a particular species and to appreciate the possible role of *Wolbachia* in transitions from bisexual to unisexual reproduction.

In the present study, we examined the prevalence (proportion of infected individuals) of *Wolbachia* infection in different populations of five *Cacopsylla* species with various life strategies, namely *C. myrtilli* (Wagner, 1947), *C. ledi* (Flor, 1861), *C. fraudatrix* Labina et Kuznetsova, 2012, *C. borealis* Nokkala et Nokkala, 2019, and *C. lapponica* Nokkala et Nokkala, 2019. The Holarctic boreo-alpine species, *C. myrtilli* and *C. ledi*, are strongly female-biased parthenogenetic species, albeit males at a very low frequency may occur in certain populations [43,44,45,46,48]. Females of these species are generally triploid with 2n = 3x = 36 + XXX, while our recent studies revealed presence of diploid females with 2n = 24 + XX among the triploids in several populations of these species [45,46]. *C. fraudatrix*, described from the Bieszczady Mountains, Poland, and *C. lapponica*, a rare alpine taxon restricted to a high-altitude open habitat, represent a truly bisexual species with 2n = 24 + XX/X(0), demonstrating nearly equal sex ratio [47,49]. Finally, a widely distributed species *C. borealis* is pentaploid, with apomictic females displaying 2n = 5x = 60 + XXXXX [47].

A key question that we address here is: are the parthenogenetic and bisexual patterns observed in different *Cacopsylla* species and even within distinct populations of the same species related to *Wolbachia* infection? If we assume this, we could expect broad distribution and high infection frequency within parthenogenetic populations/species and, on the contrary, absence or significantly lower *Wolbachia* prevalence in bisexual entities.

## 2. Materials and Methods

### 2.1. Taxon Sampling, Wolbachia Screening, and Sequencing

**Figure 1:**
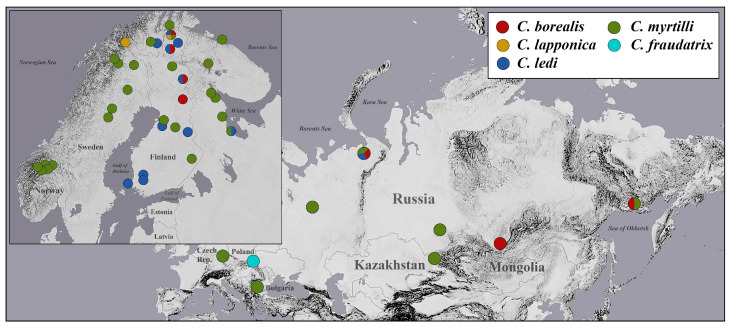
Map showing sampling localities of the analyzed specimens of *Cacopsylla*.

To examine the patterns of *Wolbachia* infection, 526 specimens belonging to five *Cacopsylla* species (*C. borealis*, *C. lapponica*, *C. myrtilli*, *C. ledi*, and *C. fraudatrix*) were collected in Finland, Sweden, Norway, Poland, Czech Republic, Bulgaria, and different regions of Russia (Figure 1, Table 1 and Table S1). Entire specimens or the head and thorax part of individual *Cacopsylla* specimens were used for DNA extraction using DNeasy Blood and Tissue Kit and QIAamp DNA Investigator Kit (Qiagen, Inc. Valencia, CA, USA) following the manufacturer’s protocols. Samples were processed at the Department of Biology of the University of Turku (Turku, Finland) and at the Department of Karyosystematics of the Zoological Institute of the Russian Academy of Sciences (Saint-Petersburg, Russia).

We screened all host specimens for *Wolbachia* infection by amplifying two genes, *Wolbachia surface protein* (*wsp*) and *16S ribosomal RNA*, commonly used as markers to detect the presence of the bacteria. We used *Wolbachia*-specific primer pairs, wsp81F/wsp691R [50] and W-Specf/W-Specr [51], amplifying ~ 550 bp fragment of the *wsp* gene and ~ 440 bp fragment of the *16S RNA* gene (actual fragment sizes depended on the individual *Wolbachia* strain), respectively. PCR reactions were carried out in 20 µL volume (1 × PCR buffer, 2.0 mM MgCl_2_, 200 μM dNTP each, 0.5 μM of forward and reverse primers, 0.5 U DreamTaq DNA Polymerase (ThermoFisher Scientific, Waltham, MA, USA), and 1 μL (ca. 50 ng) of template DNA) using the following thermal profile: initial 5 min denaturation at 95 °C followed by 40 cycles of 30 s denaturation at 95 °C, 1 min annealing at 45.5 °C (*wsp*)/49 °C (*16S*), 45 s extension at 72 °C with a final extension at 72 °C for 2 min. Each PCR reaction contained negative (PCR mix with ddH_2_O instead of DNA sample) and positive (genomic DNA of a *Wolbachia*-infected *Cacopsylla* specimen with already obtained *wsp* gene PCR product) controls. To ascertain the presence/absence of *Wolbachia*, each PCR product was checked on 1% standard agarose gel supplemented with 0.005% Midori Green Advance DNA stain (Nippon Genetics, Tokyo, Japan). After electrophoresis, the gels were inspected and photographed using GelDocEZ Imager (BioRad, Hercules, CA, USA). If a specimen was negative for one gene while positive for the other one, PCR was repeated in order to avoid the technical errors. Samples sufficiently yielding a product of the expected size for *wsp* and/or *16S RNA* genes were scored as positive for *Wolbachia*; otherwise, they were scored as negative.

PCR products were purified using QIAquick PCR Purfication Kit (Qiagen, Inc. Valencia, CA, USA) or enzymes FastAP and ExoI (Thermofisher, Waltham, MA, USA) and sent to Macrogen Europe (Amsterdam, the Netherlands) or Evrogen (Moscow, Russia) for sequencing. Obtained sequences were deposited in GenBank under the accession numbers MZ684102-MZ684135.

### 2.2. Molecular Data Analysis

The sequences were edited and aligned using Geneious 8.1.6 [52] and BioEdit 7.0.3 [53] software. The BLAST algorithm implemented in NCBI (https://blast.ncbi.nlm.nih.gov, (accessed on 20 August 2021) was used to search for sequence similarities in GenBank database with known DNA (BLASTN) sequences. We mined 68 sequences with highest percentage identity match, which we included in the phylogenetic analysis. To estimate phylogenetic relationships among *Wolbachia* alleles, a Bayesian approach was used. The analysis was performed using the MrBayes v.3.2.6 software [54] with the nucleotide substitution model GTR + G+I as suggested by jModelTest v.2.1.7 [55]. Two independent MCMC runs of 10 million generations, with four simultaneous chains (one cold and three heated) for each analysis, were performed. The sampling of trees and parameters was set to every 1000 generations. The first 10% of trees were discarded as burn-in prior to computing a consensus phylogeny and posterior probabilities. The consensus of the obtained trees was visualized using FigTree 1.3.1 (http://tree.bio.ed.ac.uk/software/figtree/, (accessed on 20 August 2021). TRACER, v.1.6 was used for summarizing the results of the Bayesian phylogenetic analysis (http://beast.bio.ed.ac.uk/Tracer, (accessed on 20 August 2021).

### 2.3. Sample Karyotyping

For Cacopsylla specimens collected in alcohol, a novel approach was developed that allows both chromosomal and molecular analyses of the same individual [44]. An individual was dissected and the abdomen was immersed in 3:1 Carnoy fixative overnight while both head and thorax remained in alcohol for subsequent DNA extraction, PCR, and sequencing. Chromosomal preparations were made, stained, and photographed following the method described earlier [44,45,46,47].

## 3. Results

### 3.1. PCR Screening and Geographical Pattern of Wolbachia Incidence

A total of 526 specimens belonging to five *Cacopsylla* species were screened for the presence of *Wolbachia* infection. Of these, 414 specimens were tested for both *wsp* and *16S* genes, 67 specimens were tested for *16S* gene only, 45 specimens were tested for *wsp* gene only. In general, screening for *wsp* and *16S* genes has shown similar patterns of infection. Only 16 specimens out of 414 ones (3.9%) tested for two genes demonstrated dissimilar results, i.e., were positive for one gene, but negative for another gene (see Table S1).

***Cacopsylla lapponica*** Nokkala et Nokkala, 2019

This is a rare, bisexually reproducing diploid species distributed in northern Fennoscandia at high altitudes above forest zone. The species has a karyotype 2n = 24 + XX/X(0) (female/male) like most species of the genus *Cacopsylla* and the superfamily Psylloidea as a whole [47].

**Figure 2:**
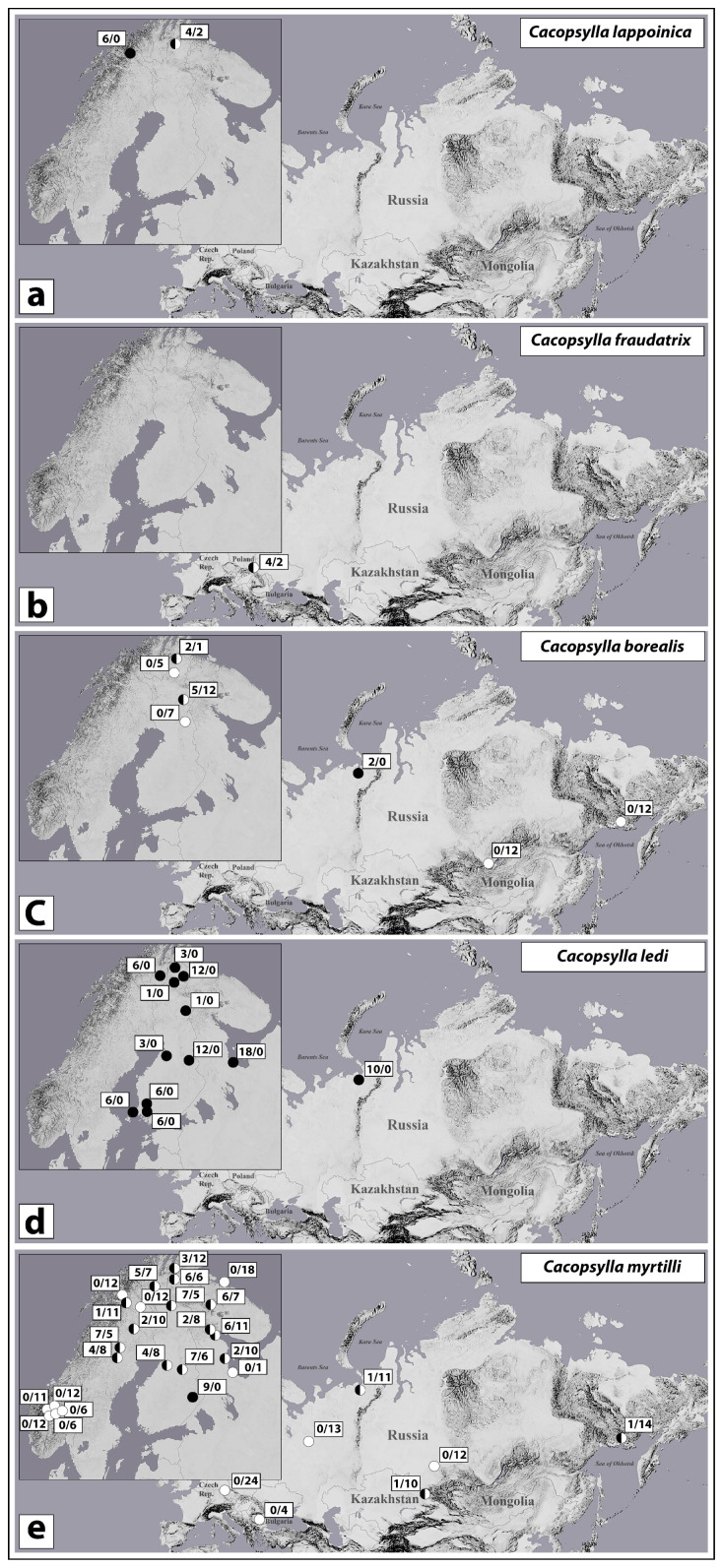
Distribution of *Wolbachia* infection in five *Cacopsylla* species (**a**–**e**). White circles indicate uninfected populations; black circles indicate completely infected populations; black-and-white circles indicate mixed populations comprising both infected and uninfected specimens. Numbers at circles indicate proportion of infected specimens (before slash) to uninfected specimens (after slash) in each population.

A total of 12 specimens (2 males and 10 females) from two geographically adjacent populations from northern Finland were studied (Figure 2a). *Wolbachia* screening for both *wsp* and *16S* genes demonstrated similar results, with only one exception. Sample CLAPP_f2 from Utsjoki, Ailigas was positive for *16S* gene, but negative for *wsp* gene. *Wolbachia* infection was predominant in both populations. In total, 10 specimens (two males and eight females) were scored positive for *Wolbachia* infection (prevalence: 83.33% [51.59–97.91%] *).

* Here and further, values in square brackets are confidential interval.

***Cacopsylla fraudatrix*** Labina et Kuznetsova, 2012

This is a bisexually reproducing diploid species, restricted in distribution to Bieszczady Mountains (Poland). The species has 2n = 24 + XX/X(0) (female/male). *C. fraudatrix* thus shares the most commonly encountered karyotype in the genus *Cacapsylla* and in the superfamily Psylloidea as a whole [49].

A total of six specimens (one male and five females) from Bieszczady population were studied (Figure 2b). Of these, four specimens (one male and three females) were screened for both *wsp* and *16S* genes; two specimens (females) were screened for *wsp* gene only. In total, four specimens (females) were scored positive for *Wolbachia* infection (prevalence: 66.67% [22.28–95.67%]).

***Cacopsylla borealis*** Nokkala et Nokkala, 2019

This is a relatively common and widespread Palaearctic species, distributed from northern Fennoscandia in the west to Magadan in the east. *C. borealis* is a pentaploid species with 2n = 5x = 60 + XXXX and apomictic parthenogenetic reproduction. No males have been recorded in *C. borealis* [47].

A total of 58 specimens (females) from seven geographically distinct populations (northern/central Finland and Russia (Vorkuta, Baikal Lake, and Magadan)) were studied for both *wsp* and *16S* genes (Figure 2c). *Wolbachia* screening demonstrated similar results except for two samples from Utsjoki (Finland), which were positive for *16S* gene but negative for *wsp* gene. *Wolbachia* screening suggested 100% infection rate in the Vorkuta (Russia) population and a moderate infection rate in the population from Heitala (Finland). The other five populations were found to be *Wolbachia*-free. In total, nine specimens were scored positive for *Wolbachia* infection (prevalence: 15.52% [7.35–27.42%]).

***Cacopsylla ledi*** (Flor, 1861)

This species is widely distributed throughout Fennoscandia, Central Europe, and Russia and occasionally forms sympatric populations with *C. borealis*. Its habitats are restricted to the temperate and alpine zones. The species is triploid (2n = 3x = 36 + XXX) and reproduces through apomictic parthenogenesis, while infrequent functional males with 2n = 24 + X(0) can be found in some populations. In populations with rare males, infrequent diploid females with 2n = 24 + XX also exist among the triploids [45].

A total of 84 specimens (52 females and 32 males) from 12 populations from Fennoscandia and northern Russia (Vorkuta) were studied (Figure 2d). Of these, 45 specimens were screened for both *wsp* and *16S* genes, 33 specimens were tested for *16S* gene only, and 6 specimens were tested for *wsp* gene only. Two females from White Sea, Russia (CLKZ_f1 and CLKZ_f8) and one male from Finnmark, Norway were positive for *16S* gene and negative for *wsp* gene. One specimen from Utsjoki, Finland (CLUH_f3) was positive for *wsp* gene but negative for *16S* gene. In total, all 84 specimens were scored positive for *Wolbachia* infection (prevalence: 100% [95.7–100%]).

***Cacopsylla myrtilli*** (Wagner, 1947)

This species is widely distributed throughout the Palaearctic, while its distribution also shows a shift towards the north and/or high altitudes. As in *C. ledi*, females of this species are usually triploid (2n = 3x = 36 + XXX) and reproduce through apomictic parthenogenesis, while rare diploids also exist. Infrequent, mainly nonfunctional, but also functional, males can be found in some populations [44,45].

A total of 366 specimens (6 males and 360 females) from 32 geographically distinct populations (Fennoscandia; Czech Republic; Bulgaria; Kazakhstan; and north, central, and east Russia) were studied (Figure 2e). Of these, 295 specimens were screened for both *wsp* and *16S* genes, 34 specimens were tested for *16S* gene only, and 37 specimens were tested for *wsp* gene only. Eight specimens were positive for *16S* gene and negative for *wsp* gene. In total, 84 specimens out of 366 studied ones (including one of the six males) were scored positive for *Wolbachia* infection (prevalence: 22.95% [18.74–27.61%]).

PCR screening revealed a complicated geographical pattern of *Wolbachia* incidence in this species. Large geographical areas, including southern and central Europe (Bulgaria, Czech Republic), southern and northwestern Norway, northern Sweden, northern Russia (Kildin Island (Barents Sea), Syktyvkar), and Siberia (Kemerovo) were *Wolbachia*-free. A low infection rates were found in Kazakhstan, Vorkuta (northern Russia), and Magadan (Russian Far East). A moderate infection rate was detected in the majority of populations from central and northern Fennoscandia. A single, completely infected population, Tohmajärvi, was found in Central Finland.

### 3.2. Wolbachia Alleles Identified in Cacopsylla Species

A 541 bp fragment of the *Wolbachia wsp* gene was sequenced for 34 infected specimens of five host *Cacopsylla* species, *C. borealis*, *C. lapponica*, *C. ledi*, *C. myrtilli*, and *C. fraudatrix* (Table 2). 

Sequencing revealed three *wsp* alleles among samples from four host species, *C. borealis*, *C. lapponica*, *C. ledi*, and *C. myrtilli*. In *C. fraudatrix,* at least two alternative *Wolbachia* alleles were found, however the presence of heteroplasmy, indicated by double peaks on the chromatograms, does not allow unambiguously assigning sequences to a particular *wsp* allele (for the identified alleles, see Table 3).

**Figure 3:**
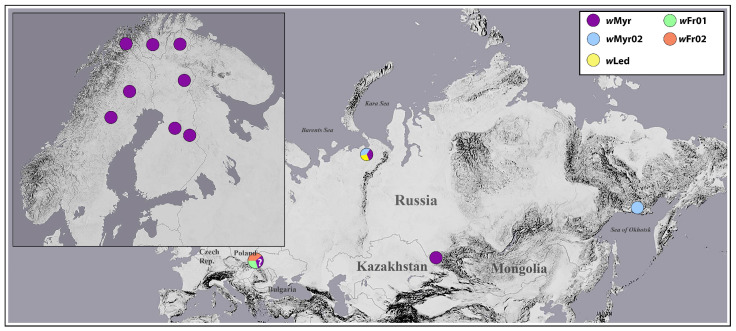
Map showing geographical distribution of revealed *Wolbachia wsp* alleles.

BLAST search ascertained that the obtained sequences were unique, showing that they belonged to the supergroup B (grouping according to [56]). Comparison with *Wolbachia* sequences deposited in the public databases GenBank (http://www.ncbi.nlm.nih.gov, (accessed on 20 August 2021) and PubMLST-*Wolbachia* (https://pubmlst.org/organisms/wolbachia-spp, (accessed on 01 September 2021) revealed that *Wolbachia* alleles isolated from *Cacopsylla* hosts were most similar to *wsp* allele 639 found in lycaenid butterflies [57,58], differing from the latter in 3–4 nucleotide substitutions only. Most of the specimens analyzed (24 out of 30) of the host species *C. myrtilli*, *C. borealis*, *C. lapponica*, and *C. ledi* harbored the major *wsp* allele, *w*Myr01 (allele names follow the earlier accepted abbreviation style [50,59]). Most likely, this allele is also present in *C. fraudatrix* specimens CFR_f5, CFR_f7, and CFR_f9. However, cloning procedure is required to confirm this suggestion. The only *Wolbachia*-positive specimen of *C. myrtilli* (CMMAG_f10) from Magadan (out of the 15 specimens analyzed) differed from the most common and widespread allele *w*Myr01 in one nucleotide substitution (transition A = >G) at site 498. The same substitution was found in one *C. borealis* specimen (CV_f11) from Vorkuta. This allele was designated as *w*Myr02. The other specimen of *C. borealis* from Vorkuta (CBV_f1) (out of the two specimens analyzed) possessed a nucleotide substitution (transversion G = >C) at site 499. The same transversion was found in three *C. ledi* specimens from Vorkuta (CLV_f2, CLV_f3, CLV_f6). This allele was designated as *w*Led. *C. fraudatrix* specimen CFR_f8 differed from all other samples by a nucleotide substitution C = >A at site 495. In addition, it displayed intra-individual polymorphism at site 360, indicating presence of two alleles not yet found in other *Cacopsylla* species and designated here as *w*Fr01 and *w*Fr02, respectively. Two polymorphic positions (C/A at sites 360 and 495) found in *C. fraudatrix* samples CFR_f5, CFR_f7, and CFR_f9 did not allow unambiguously assigning sequences to any *wsp* allele. Most likely, these specimens were infected by two or three *Wolbachia* alleles (*w*Myr01, *w*Fr02, and/or *w*Fr01). Geographical distribution of revealed *Wolbachia* alleles is shown in Figure 3.

### 3.3. Phylogenetic Inferences 

**Figure 4:**
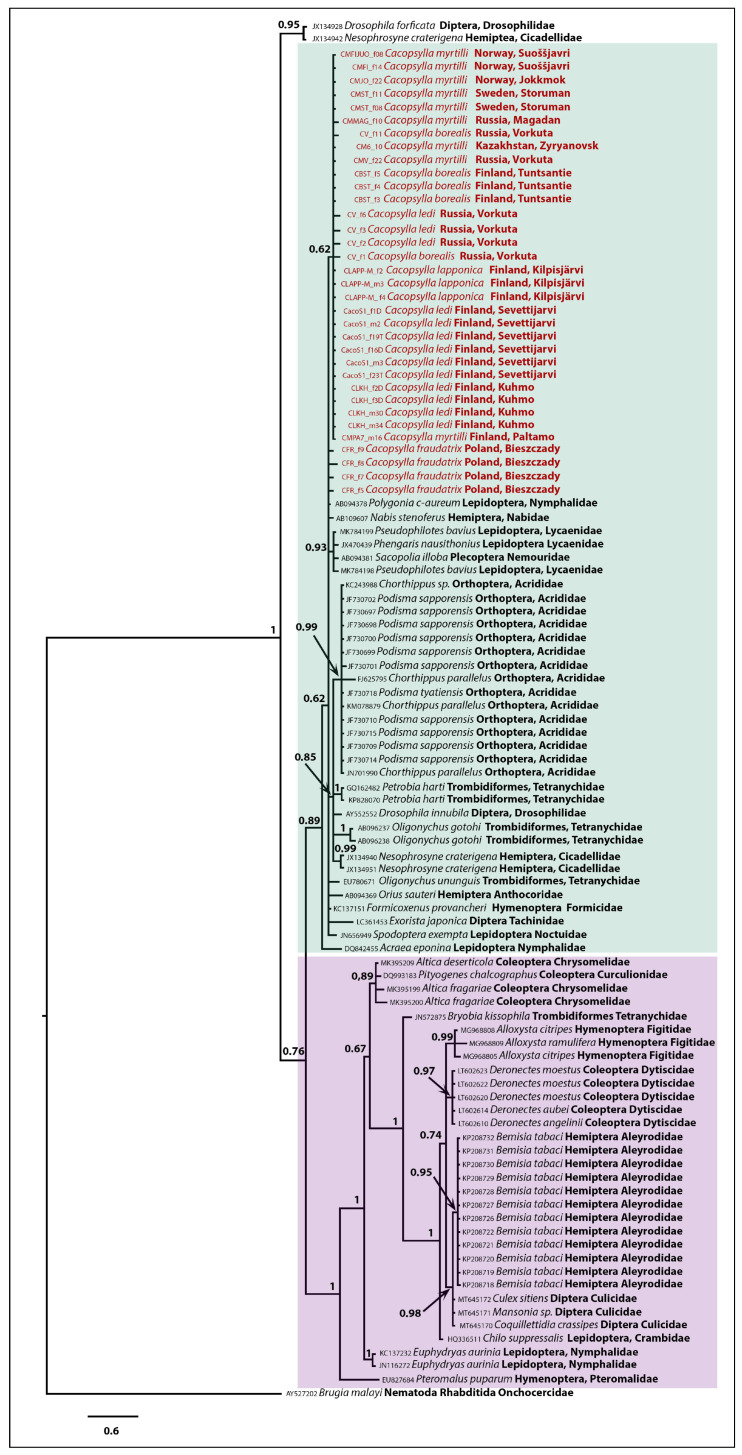
The Bayesian tree of analyzed *Wolbachia* samples. Numbers at nodes indicate Bayesian posterior probability. Scale bar = 0.6 substitutions. Revealed *Wolbachia* clusters are highlighted in violet and green.

We used the 68 most similar *Wolbachia wsp* sequences with known host species mined from GenBank to infer the *Wolbachia* phylogeny. *Wolbachia wsp* sequence of nematode *Brugia malayi* (Brug, 1927) (GenBank accession number AY527202) belonging to the supergroup D was included in our analysis as an outgroup to root the phylogeny. Bayesian analysis recovered two clades (Figure 4) that received high (clade II, BS = 1) and moderate (clade I, BS = 0.89) support. All *wsp* sequences of five host *Cacopsylla* species obtained in the present study clustered within clade I together with *Wolbachia* strains from different insect orders (Lepidoptera, Plecoptera, Orthoptera, Hemiptera, Hymenoptera, Diptera) and acariform mites (Trombidiformes). Maximum p-distances between samples constituting clade I were as high as 2.92%. The second clade comprised *Wolbachia* strains found in Coleoptera, Hymenoptera, Hemiptera, Lepidoptera, Diptera (Insecta), and Trombidiformes (Arachnida). Genetic distances between *Wolbachia* strains of the clade II and those found in *Cacopsylla* ranged from 5.29% to 7.48%.

## 4. Discussion

### 4.1. Wolbachia Infection in Hemiptera

Numerous reports on *Wolbachia* in different arthropod taxa have clearly shown that the infection is widespread across this group of animals. However, recent studies have suggested that, to date, less than 1% of all existing *Wolbachia* strains have been characterized [60]. The most comprehensive molecular data on *Wolbachia* sequences and allelic profiles can be found on the PubMLST-*Wolbachia* database, available at http://pubmlst.org/wolbachia/, (accessed on 01 September 2021) [61]. The data representation in the database is strongly biased to five insect orders (Lepidoptera, Coleoptera, Hemiptera, Hymenoptera, and Diptera), which account for 79% of the total *Wolbachia* strains deposited. Despite the fact that Hemiptera is one of the most represented orders (297 (14.7%) out of over 2000 *Wolbachia* strains deposited in PubMLST-*Wolbachi*a (by September 2021) belong to Hemiptera), most hemipteran families and genera have not yet been examined. Moreover, the majority of the *Wolbachia* strains were characterized in Hemiptera species inhabiting Asia, Africa, South America, and Australasia, while only one record comes from Europe. For the family Psyllidae, only 26 *Wolbachia* strains are reported in PubMLST-*Wolbachi*a (none of these belong to the genus *Cacopsylla*)—eight were isolated from *Diaphorina citri* and one was isolated from *Heteropsylla* sp., while for 17 isolated strains the host genus and the host species were not determined. Nevertheless, a number of studies dedicated to *Cacopsylla* associations with various bacterial endosymbionts [62,63], including *Wolbachia* [64,65], have been published over the past decade. It should be noted, that most of the published investigations contain data on a single (or very low number) of species, in which usually only a few individuals were tested. Testing only a small number of specimens and/or screening single *Wolbachia*-specific genes could obscure our understanding of the presence and actual rate of *Wolbachia* infection, and, therefore, the impact of *Wolbachia* on biology and evolution of host species. Firstly, it significantly increases the possibility of occasionally picking uninfected specimens from a pool of infected and uninfected individuals, especially if prevalence rates are low. Low infection rate was reported, for instance, for fruit fly *Bactocera dorsalis* (Hendel, 1912) (Diptera: Tephritidae), where screening of 1500 individuals revealed infection frequencies ranging from 0% to 3% [66]. Secondly, frequency of infection may vary significantly (from 0% to 100%) between different populations of the same species, as it was shown for fruit flies [67], planthoppers [68], and for *Cacopsylla* in the present study. Thirdly, species might also be erroneously classified as uninfected due to low-titer infections that are not detected by common PCR screening or extremely weak/unsuccessful amplification if nucleotide substitutions in the regions targeted by *Wolbachia*-specific primers are present, resulting in a significant decrease of primer efficiency. Finally, some *Wolbachia* genes can be incorporated into the host nuclear genome, leading to false positive PCR results. Thus, in order to improve estimates of infection frequencies and more accurately assess the incidence of *Wolbachia* and its influence on host species, it is crucial to use several *Wolbachia*-specific molecular markers and to include a large-scale sampling dataset in the analysis.

### 4.2. Patterns of Wolbachia Infection in Cacopsylla

Our study is the first to report patterns and diversity of *Wolbachia* infection in five jumping plant-lice species of the genus *Cacopsylla* based on geographically wide sampling, using PCR assays for the *Wolbachia*-specific *wsp* and *16S* genes. In general, screening for *wsp* and *16S* genes has shown a similar pattern of infection in the studied species. Only 16 specimens (3.9%) out of 414 tested for two genes demonstrated contradictory results, with 15 cases positive for *16S* gene and negative for *wsp* gene. We can assume that *Wolbachia* persists in these specimens at a low density and, thus, conventional PCR-screening does not detect infection if the used primers are not sensitive enough. Recent surveys have suggested that low-titer *Wolbachia* infection within arthropod hosts is more common than considered previously [68,69]. Alternatively, the absence of amplification using *wsp* primers can be explained by primer/template mismatch in the region targeted by *Wolbachia*-specific primers, leading to unsuccessful PCR amplification. 

The studied *Cacopsylla* species displayed a complex pattern of *Wolbachia* infection. They included both infected and uninfected populations and different *wsp* gene alleles, which corresponded to their geographical distribution. A total of five *wsp* alleles were revealed. The geographically widely distributed *w*Myr01 is shared by *Wolbachia* infecting *C. myrtilli, C. borealis*, *C. lapponica*, *C. ledi*, and, probably, *C. fraudatrix* (see Section 3.2. in Results). This suggests either common origin of the infection or the transmission of this allele between the species. Allele *w*Myr02 was encountered in *C. myrtilli* only, being found in two geographically distinct populations from Vorkuta and Magadan. Allele *w*Led was encountered in *C. ledi* and *C. borealis*, being found only in Vorkuta populations. Thus, three alleles, *w*Myr01, *w*Myr02, and *w*Led, sympatrically co-exist in Vorkuta. Alleles *w*Fr01 and *w*Fr02 were found only in *C. fraudatrix*, which was the only species showing intra-individual allele polymorphism, suggesting coinfection by genetically different *Wolbachia*. We can speculate that allele *w*Fr01 first appeared as a result of a single substitution in allele *w*Myr01; subsequently, substitution in allele *w*Fr01 led to evolving *w*Fr02. The data obtained suggest that scenarios of *Wolbachia* infection in *C. fraudatrix* and other *Cacopsylla* species were different.

*Wolbachia* was predominant in diploid bisexual species *C. lapponica* and *C. fraudatrix* (infection rate 83% and 67%, respectively) and totally infected triploid parthenogenetic species *C. ledi.* Unlike *C. ledi*, “female-only” pentaploid *C. borealis* demonstrated a very low rate of infection (15%), with several *Wolbachia*-free populations. The most complicated pattern of *Wolbachia* incidence was observed in triploid parthenogenetic *C. myrtilli*. Different populations of this species showed either (1) total infection, (2) a moderate rate of infection, (3) a low rate of infection, or (4) absence of infection, respectively. Some *Wolbachia*-free populations were found to be surrounded by moderately infected populations, e.g., Soppero and Björkliden in northern Sweden, and Kildin Island in Russia. These populations were probably formed by *Wolbachia*-free founder specimens. A quite different scenario can be suggested to explain existence of vast *Wolbachia*-free areas encompassing southern Norway, central and southeastern Europe (Czech Republic, Bulgaria), the Polar Ural Mountains, and Siberia (Russia). We hypothesize that *Cacopsylla* species inhabiting these areas originated in *Wolbachia*-free refugia during the post-glacial period.

Phylogenetic analysis revealed a high rate of similarity between *wsp* sequences of five host *Cacopsylla* species, with *Wolbachia* strains infecting insect orders Lepidoptera, Plecoptera, Orthoptera, Hemiptera, Hymenoptera, Diptera, and acariform mites (Trombidiformes). Sharing similar *Wolbachia* alleles between *Cacopsylla* and members of other arthropods can be explained neither by common origin nor hybridization, with the most likely explanation being that it is a result of horizontal transfer. Particular *Wolbachia* strains can be transferred to phylogenetically distant arthropods either through shared parasitoids [70] or, in plant-sup feeders, through their saliva [71,72,73].

## 5. Conclusions

Our study is the first attempt of large-scale investigation aimed at a detailed analysis of presence, prevalence, geographical distribution, and molecular characterization of *Wolbachia* infection in the species-rich hemipteran genus *Cacopsylla* (Hemiptera, Psylloidea). The genus comprises species with different reproductive strategies (bisexual and parthenogenetic) and ploidy levels (diploids, triploids, and pentaploids). One of the well-known effects of *Wolbachia* is male killing, leading to a prevalence of females or complete elimination of males in the certain populations of the host species and a subsequent shift to parthenogenetic reproduction. We tested the assumption that a variety of reproductive strategies observed in distinct *Cacopsylla* species and the existence of “female-only” populations or populations heavily biased towards females are the consequences of *Wolbachia* infection. Our survey did not reveal significant differences in infection frequency between parthenogenetic and bisexual populations or between males and females within bisexual populations. We suggest that the evolution of various reproductive strategies in *Cacopsylla* is not attributable to an effect of *Wolbachia* and was probably driven by some other, most likely environmental factors, which have not yet been studied. At the same time, complicated phylogeographic patterns of *Wolbachia* infection were observed in the genus *Cacopsylla*. We found that *Wolbachia* infected all five studied *Cacopsylla* species, reflecting the significant role of the endosymbiont in their biology. We conclude that our survey in the context of complex temporal and spatial patterns of *Wolbachia* infection represents a potential model for future research and provides insights into the factors impacting *Wolbachia* and its interactions with psyllids and insects as a whole.

## Figures and Tables

**Table 1:** Sampling localities.

Table	Samples	Locality
*Cacopsylla lapponica*	2♂ + 4♀	FINLAND, Lapland province, Kilpisjärvi, Pikku-Malla, 69°08′56″ N; 20°44′20″ E, h = 610 m, 27.07.2014, S. Nokkala & Ch. Nokkala leg.
	6♀	FINLAND, Lapland province, Utsjoki, Ailigas, 69°53′51″ N; 27°03′32″ E, 28.07.2020, S. Nokkala & Ch. Nokkala leg.
*Cacopsylla fraudatrix*	1♂ + 5♀	POLAND, Bieszczady Mts, Wielka Rawka, 49°06′ N; 22°35′ E, 07.08.2009, V. Kuznetsova & A. Maryańska-Nadachowska leg.
*Cacopsylla borealis*	17♀	FINLAND, Lapland province, Tuntsantie, 67°18′11″ N; 29°16′18″ E, 01.08.2019, S. Nokkala & Ch. Nokkala leg.
	5♀	FINLAND, Lapland province, Inari, Pitkävuono, 68°58′56″ N; 26°57′18″ E, 16.08.2017, S. Nokkala & Ch. Nokkala leg.
	3♀	FINLAND, Lapland province, Utsjoki, Hietala 69°51′06″ N; 27°00′34″ E, 15.08.2017, S. Nokkala & Ch. Nokkala leg.
	7♀	FINLAND, Kuusamo, Kantojoki, 66°14′23″ N; 29°09′15″ E, 02.08.2019, S. Nokkala & Ch. Nokkala leg.
	12♀	RUSSIA, Magadan region, vic. Ola vill., 59°34′24″ N; 150°46′04″ E, 22.07.2020, Yu. Marusik & D. Berman leg.
	2♀	RUSSIA, Komi Republic, Vorkuta city, 67°27′34″ N; 63°59′01″ E, 06.08.2013, N. Khabazova & M. Mandelshtam leg.
	12♀	RUSSIA, Irkutsk region, Lake Baikal, vic. Bolshye Koty vill. 51°54′25″ N; 105°04′14″ E, 21.07.2007, E. Labina leg.
*Cacopsylla ledi*	2♂ + 10♀	FINLAND, Lapland province, Sevettijärvi, 69°12′58″ N; 27°52′14″ E, 17.08.2017, S. Nokkala & Ch. Nokkala leg.
	3♀	FINLAND, Oulu province, Siikajoki, 64°39′32″ N; 25°19′33″ E, 10.08.2018, S. Nokkala & Ch. Nokkala leg.
	6♂ + 6♀	FINLAND, Oulu province, Kuhmo, Syväjärvi, 64°11′40″ N; 29°57′49″ E, 04.08.2019, S. Nokkala & Ch. Nokkala leg.
	6♂	FINLAND, Western Finland province, Yläne, 60°53′10″ N; 22°26′41″ E, 15.08.2019, S. Nokkala & Ch. Nokkala leg.
	6♂	FINLAND, Western Finland province, Pöytyä, Lammenrahka, 60°44′37″ N; 22°25′32″ E, 16.08.2018, S. Nokkala & Ch. Nokkala leg.
	6♂	FINLAND, Western Finland province, Kustavi, 60°39′20″ N; 21°18′12″ E, 25.08.2019, S. Nokkala & Ch. Nokkala leg.
	1♀	FINLAND, Lapland province, Inari, Pitkävuono, 68°58′56″ N; 26°57′18″ E, 16.08.2017, S. Nokkala & Ch. Nokkala leg.
	3♀	FINLAND, Lapland province, Utsjoki, Hietala 69°51′06″ N; 27°00′34″ E, 15.08.2017, S. Nokkala & Ch. Nokkala leg.
	1♀	FINLAND, Lapland province, Tuntsantie, 67°18′11″ N; 29°16′18″ E, 01.08.2019, S. Nokkala & Ch. Nokkala leg.
	6♂	NORWAY, Troms og Finnmark county, Mohkkejogas, 69°26′35″ N; 25°11′36″ E, 26.07.2020, S. Nokkala & Ch. Nokkala leg.
	18♀	RUSSIA, Karelia Republic, White Sea, vic. Kolezma vill., 64°14′46″ N 35°48′49″ E, 30.09.2020, V. Kuznetsova & P. Strelkov leg.
	10♀	RUSSIA, Komi Republic, Vorkuta city, 67°27′34″ N; 63°59′01″ E, 06.08.2013, N. Khabazova & M. Mandelshtam leg.
*Cacopsylla myrtilli*	1♂ + 12♀	FINLAND, Kainuu province, Paltamo district, S of Törmänmäki, 64°33′28″ N; 27°43′41″ E, 16.07.2009, S. Nokkala & Ch. Nokkala leg.
	1♂ + 14♀	FINLAND, Lapland province, Utsjoki, Ailigas, 69°53′51″ N; 27°03′32″ E, 16.08.2017, S. Nokkala & Ch. Nokkala leg.
	12♀	FINLAND, Lapland province, Utsjoki, Hietala 69°51′06″ N; 27°00′34″ E, 11.08.2012, S. Nokkala & Ch. Nokkala leg.
	12♀	FINLAND, Lapland province, Sodankylä, Puisuvanto, 67°46′52″ N; 26°46′09″ E, 03.08.2018, S. Nokkala & Ch. Nokkala leg.
	12♀	FINLAND, Oulu province, Liminka, 64°43′53″ N; 25°23′04″ E, 04.08.2009, S. Nokkala & Ch. Nokkala leg.
	9♀	FINLAND, Eastern Finland province, Tohmajärvi, 62°23′12″ N; 30°19′46″ E, 12.08.2012, S. Nokkala & Ch. Nokkala leg.
	12♀	SWEDEN, Swedish Lapland province, Abisko, Lapporten, 68°19′14″ N; 18°51′05″ E, h = 570 m, 26.07.2014, S. Nokkala & Ch. Nokkala leg.
	12♀	SWEDEN, Swedish Lapland province, Björkliden, Fjället, 68°24′32″ N; 18°39′55″ E, 03.08.2009, S. Nokkala & Ch. Nokkala leg.
	12♀	SWEDEN, Norbothnia province, Soppero, 68°00′39″ N; 21°39′25″ E, 10.08.2012, S. Nokkala & Ch. Nokkala leg.
	12♀	SWEDEN, Norbothnia province, Jokkmok, 66°35′36″ N; 19°49′20″ E, 08.08.2012, S. Nokkala & Ch. Nokkala leg.
	12♀	SWEDEN, Westrobothnia province, Sorsele, 65°29′07″ N; 17°33′36″ E, 17.08.2010, S. Nokkala & Ch. Nokkala leg.
	12♀	SWEDEN, Westrobothnia province, Storuman, 65°05′16″ N; 17°06′51″ E, 08.08.2012, S. Nokkala & Ch. Nokkala leg.
	12♀	NORWAY, Troms og Finnmark county, Suoššjavri, 69°22′11″ N; 24°18′20″ E, 18.08.2011, S. Nokkala & Ch. Nokkala leg.
	6♀	NORWAY, Innlandet county, Sjoa, Kringlothaugen mt., 61°43′06″ N; 09°22′40″ E, h = 700 m, 01.08.2009, S. Nokkala & Ch. Nokkala leg.
	6♀	NORWAY, Innlandet county, Sjoa, Kvernbrusaetrin, 61°42′27″ N; 09°19′25″ E, h = 1100 m, 01.08.2009, S. Nokkala & Ch. Nokkala leg.
	12♀	NORWAY, Innlandet county, Sjoa, Stålane, 61°41′15″ N; 09°14′27″ E, h = 1100 m, 01.08.2009, S. Nokkala & Ch. Nokkala leg.
	12♀	NORWAY, Innlandet county, Sjoa, Rudihoe, 61°46′27″ N; 09°17′16″ E, h = 1100 m, 01.08.2009, S. Nokkala & Ch. Nokkala leg.
	11♀	NORWAY, Innlandet county, Sjoa, Rindhovda, 61°43′05″ N; 09°05′12″ E, h = 1080 m, 16.08.2010, S. Nokkala & Ch. Nokkala leg.
	18♀	RUSSIA, Murmansk region, Barents Sea, Kildin Island, 69°19′58″ N; 34°23′43″ E, 05.08.2016, P. Strelkov leg.
	3♂ + 10♀	RUSSIA, Murmansk region, Laplandsky Natural Reserve, 68°07′ N; 32°27′ E, 01.08.2019, A. Polevoi leg.
	1♀	RUSSIA, Karelia Republic, White Sea, vic. Kolezma vill., 64°14′46″ N 35°48′49″ E, 30.09.2020, V. Kuznetsova & P. Strelkov leg.
	10♀	RUSSIA, Karelia Republic, Louchskiy district, “Belomorskaya″ Research Station, 66°17′58″ N; 33°37′18″ E, 29.08.2019, G. Paskerova leg.
	17♀	RUSSIA, Karelia Republic, White Sea, Sredniy Island, 66°17′28″ N; 33°39′06″ E, 21.08.2017, G. Paskerova leg.
	12♀	RUSSIA, Karelia Republic, vic. Kem′ city, 2017.
	15♀	RUSSIA, Magadan region, vic. Ola vill., 59°34′24″ N; 150°46′04″ E, 22.07.2020, Yu. Marusik & D. Berman leg.
	12♀	RUSSIA, Komi Republic, Vorkuta city, 67°27′34″ N; 63°59′01″ E, 06.08.2013, N. Khabazova & M. Mandelshtam leg.
	13♀	RUSSIA, Komi Republic, 2 km W of Syktyvkar city, 61°38′57″ N; 50°44′09″ E, 02.09.2020, A. Zinovieva leg.
	12♀	RUSSIA, Kemerovo region, Kemerovskiy district, vic. Voskresenka vill., 55°19′ N; 86°48′ E, July 2019. A. Polevoi leg.
	1♂ + 10♀	KAZAKHSTAN, Zyryanovsky district, ca. 30 km N of Zyryanovsk city, 50°00′05″ N; 84°13′32″ E, 11.07.2012, V. Lukhtanov leg.
	12♀	CZECH REPUBLIC, Karlovy Vary region, Kleiner Kranichsee Natural Reserve, 50°24′56″ N; 12°40′30″ E, h = 925 m, 28.07.2020, I. Malinovsky leg.
	12♀	CZECH REPUBLIC, Karlovy Vary region, Kleiner Kranichsee Natural Reserve, 50°23′33″ N; 12°37′48″ E, h = 915 m, 29.07.2020, I. Malinovsky leg.
	4♀	BULGARIA, Kyustendil province, Rila, Stara planina Mts., 42°06′ N; 23°33′ E, 17.08.2020, I. Gjonov leg.

**Table 2:** List of specimens sequenced for *Wolbachia wsp* gene.

Host Taxon	Sample ID	GB Accession No.	Sex	Allele	Locality
*Cacopsylla lapponica*	CLAPP-M_f2	MZ684116	♀	wMyr01	FINLAND, Kilpisjärvi, 69°08′56″ N; 20°44′20″ E
*Cacopsylla lapponica*	CLAPP-M_m3	MZ684117	♀	wMyr01	FINLAND, Kilpisjärvi, 69°08′56″ N; 20°44′20″ E
*Cacopsylla lapponica*	CLAPP-M_f4	MZ684118	♂	wMyr01	FINLAND, Kilpisjärvi, 69°08′56″ N; 20°44′20″ E
*Cacopsylla fraudatrix*	CFR_f5	MZ684132	♀	*	POLAND, Bieszczady 49°06′ N; 22°35′ E
*Cacopsylla fraudatrix*	CFR_f7	MZ684133	♀	*	POLAND, Bieszczady 49°06′ N; 22°35′ E
*Cacopsylla fraudatrix*	CFR_f8	MZ684134	♀	wFr01 wFr02	POLAND, Bieszczady 49°06′ N; 22°35′ E
*Cacopsylla fraudatrix*	CFR_f9	MZ684135	♀	*	POLAND, Bieszczady 49°06′ N; 22°35′ E
*Cacopsylla borealis*	CBST_f3	MZ684111	♀	wMyr01	FINLAND, Tuntsantie, 67°18′11″ N; 29°16′18″ E
*Cacopsylla borealis*	CBST_f4	MZ684112	♀	wMyr01	FINLAND, Tuntsantie, 67°18′11″ N; 29°16′18″ E
*Cacopsylla borealis*	CBST_f5	MZ684113	♀	wMyr01	FINLAND, Tuntsantie, 67°18′11″ N; 29°16′18″ E
*Cacopsylla borealis*	CV_f11	MZ684114	♀	wMyr02	RUSSIA, Vorkuta 67°27′34″ N; 63°59′01″ E
*Cacopsylla borealis*	CV_f1	MZ684115	♀	wLed	RUSSIA, Vorkuta 67°27′34″ N; 63°59′01″ E
*Cacopsylla ledi*	CacoS1_f1D	MZ684119	♀	wMyr01	FINLAND, Sevettijärvi, 69°12′58″ N; 27°52′14″ E
*Cacopsylla ledi*	CacoS1_f16D	MZ684120	♀	wMyr01	FINLAND, Sevettijärvi, 69°12′58″ N; 27°52′14″ E
*Cacopsylla ledi*	CacoS1_f19T	MZ684121	♀	wMyr01	FINLAND, Sevettijärvi, 69°12′58″ N; 27°52′14″ E
*Cacopsylla ledi*	CacoS1_f23T	MZ684122	♀	wMyr01	FINLAND, Sevettijärvi, 69°12′58″ N; 27°52′14″ E
*Cacopsylla ledi*	CacoS1_m2	MZ684123	♂	wMyr01	FINLAND, Sevettijärvi, 69°12′58″ N; 27°52′14″ E
*Cacopsylla ledi*	CacoS1_m3	MZ684124	♂	wMyr01	FINLAND, Sevettijärvi, 69°12′58″ N; 27°52′14″ E
*Cacopsylla ledi*	CLKH_f2D	MZ684125	♀	wMyr01	FINLAND, Kuhmo, 64°11′40″ N; 29°57′49″ E
*Cacopsylla ledi*	CLKH_f3D	MZ684126	♀	wMyr01	FINLAND, Kuhmo, 64°11′40″ N; 29°57′49″ E
*Cacopsylla ledi*	CLKH_m30	MZ684127	♂	wMyr01	FINLAND, Kuhmo, 64°11′40″ N; 29°57′49″ E
*Cacopsylla ledi*	CLKH_m34	MZ684128	♂	wMyr01	FINLAND, Kuhmo, 64°11′40″ N; 29°57′49″ E
*Cacopsylla ledi*	CLV_f2	MZ684129	♀	wLed	RUSSIA, Vorkuta 67°27′34″ N; 63°59′01″ E
*Cacopsylla ledi*	CLV_f3	MZ684130	♀	wLed	RUSSIA, Vorkuta 67°27′34″ N; 63°59′01″ E
*Cacopsylla ledi*	CLV_f6	MZ684131	♀	wLed	RUSSIA, Vorkuta 67°27′34″ N; 63°59′01″ E
*Cacopsylla myrtilli*	CMFIJUO_f08	MZ684102	♀	wMyr01	NORWAY, Suoššjavri, 69°22′11″ N; 24°18′20″ E
*Cacopsylla myrtilli*	CMFI_f14	MZ684103	♀	wMyr01	NORWAY, Suoššjavri, 69°22′11″ N; 24°18′20″ E
*Cacopsylla myrtilli*	CMPAL_m16	MZ684104	♂	wMyr01	FINLAND, Paltamo, 64°33′28″ N; 27°43′41″ E
*Cacopsylla myrtilli*	CMST_f8	MZ684105	♀	wMyr01	SWEDEN, Storuman, 65°05′16″ N; 17°06′51″ E
*Cacopsylla myrtilli*	CMST_f11	MZ684106	♀	wMyr01	SWEDEN, Storuman, 65°05′16″ N; 17°06′51″ E
*Cacopsylla myrtilli*	CMJO_f22	MZ684107	♀	wMyr01	SWEDEN, Jokkmok, 66°35′36″ N; 19°49′20″ E
*Cacopsylla myrtilli*	CMV_f22	MZ684108	♀	wMyr01	RUSSIA, Vorkuta 67°27′34″ N; 63°59′01″ E
*Cacopsylla myrtilli*	CM6.10	MZ684109	♀	wMyr01	KAZAKHSTAN, Zyryanovsk, 50°00′05″ N; 84°13′32″ E
*Cacopsylla myrtilli*	CMMAG_f10	MZ684110	♀	wMyr02	RUSSIA, Magadan 59°34′24″ N; 150°46′04′′ E

The asterisk (*) indicates samples that cannot be attributed to any *Wolbachia* allele due to presence of more than one heterogeneity in *wsp* gene sequences.

**Table 3:** Variable sites of the studied *Wolbachia wsp* gene fragment among the 34 samples sequenced.

Allele	Nucleotide Position			
	360	495	498	499
*w*Myr01	C	C	A	G
*w*Myr02	C	C	**G**	G
*w*Led	C	C	A	**C**
*w*Fr01	C	**A**	A	G
*w*Fr02	**A**	**A**	A	G

## Supplementary Materials

The following are available online at https://www.mdpi.com/article/10.3390/insects12100853/s1, Table S1: List of studied material.
