# Supplementary material for: The Incidence of Wolbachia Bacterial Endosymbiont in Bisexual and Parthenogenetic Populations of the Psyllid Genus Cacopsylla (Hemiptera, Psylloidea)
Source: Insects. 2021 Sep 22;12(10):853. doi: 10.3390/insects12100853 (PMC8540236; doi:10.3390/insects12100853)
Supplement: Supplementary file 1 [file insects-12-00853-s001.zip › insects-1389803-supplementary.pdf]

Table S1. List of studied material.

Taxon	Sample ID	Sex	*Wolbachia 16S*	*Wolbachia wsp*	2n	Locality
C. lapponica	CLAPP-M_m1	m	+	+	24+X0	FINLAND, Lapland province, Kilpisjärvi, Pikku-Malla, 69°08'56"N; 20°44'20"E, h = 610 m, 27.07.2014, S. Nokkala & Ch. Nokkala leg.
C. lapponica	CLAPP-M_m3	m	+	+	24+X0	FINLAND, Lapland province, Kilpisjärvi, Pikku-Malla, 69°08'56"N; 20°44'20"E, h = 610 m, 27.07.2014, S. Nokkala & Ch. Nokkala leg.
C. lapponica	CLAPP-M_f2	f	+	+	24+XX	FINLAND, Lapland province, Kilpisjärvi, Pikku-Malla, 69°08'56"N; 20°44'20"E, h = 610 m, 27.07.2014, S. Nokkala & Ch. Nokkala leg.
C. lapponica	CLAPP-M_f4	f	+	+	24+XX	FINLAND, Lapland province, Kilpisjärvi, Pikku-Malla, 69°08'56"N; 20°44'20"E, h = 610 m, 27.07.2014, S. Nokkala & Ch. Nokkala leg.
C. lapponica	CLAPP-M_f5	f	+	+		FINLAND, Lapland province, Kilpisjärvi, Pikku-Malla, 69°08'56"N; 20°44'20"E, h = 610 m, 27.07.2014, S. Nokkala & Ch. Nokkala leg.
C. lapponica	CLAPP-M_f6	f	+	+		FINLAND, Lapland province, Kilpisjärvi, Pikku-Malla, 69°08'56"N; 20°44'20"E, h = 610 m, 27.07.2014, S. Nokkala & Ch. Nokkala leg.
C. lapponica	CLAPP_f1	f	+	+		FINLAND, Lapland province, Utsjoki, Ailigas, 69°53'51"N; 27°03'32"E, 28.07.2020, S. Nokkala & Ch. Nokkala leg.
C. lapponica	CLAPP_f2	f	+	-		FINLAND, Lapland province, Utsjoki, Ailigas, 69°53'51"N; 27°03'32"E, 28.07.2020, S. Nokkala & Ch. Nokkala leg.
C. lapponica	CLAPP_f3	f	-	-		FINLAND, Lapland province, Utsjoki, Ailigas, 69°53'51"N; 27°03'32"E, 28.07.2020, S. Nokkala & Ch. Nokkala leg.
C. lapponica	CLAPP_f4	f	+	+		FINLAND, Lapland province, Utsjoki, Ailigas, 69°53'51"N; 27°03'32"E, 28.07.2020, S. Nokkala & Ch. Nokkala leg.
C. lapponica	CLAPP_f5	f	+	+		FINLAND, Lapland province, Utsjoki, Ailigas, 69°53'51"N; 27°03'32"E, 28.07.2020, S. Nokkala & Ch. Nokkala leg.
C. lapponica	CLAPP_f6	f	-	-		FINLAND, Lapland province, Utsjoki, Ailigas, 69°53'51"N; 27°03'32"E, 28.07.2020, S. Nokkala & Ch. Nokkala leg.
C. fraudatrix	CFR_SP_m	m	-	-		POLAND, Bieszczady Mts, Wielka Rawka, 49º06'N; 22º35'E, 07.08.2009, V. Kuznetsova & A. Maryańska-Nadahowska leg.
C. fraudatrix	CFR_f1	f	n\a	-		POLAND, Bieszczady Mts, Wielka Rawka, 49º06'N; 22º35'E, 07.08.2009, V. Kuznetsova & A. Maryańska-Nadahowska leg.
C. fraudatrix	CFR_f5	f	n\a	+		POLAND, Bieszczady Mts, Wielka Rawka, 49º06'N; 22º35'E, 07.08.2009, V. Kuznetsova & A. Maryańska-Nadahowska leg.
C. fraudatrix	CFR_f7	f	+	+		POLAND, Bieszczady Mts, Wielka Rawka, 49º06'N; 22º35'E, 07.08.2009, V. Kuznetsova & A. Maryańska-Nadahowska leg.
C. fraudatrix	CFR_f8	f	+	+		POLAND, Bieszczady Mts, Wielka Rawka, 49º06'N; 22º35'E, 07.08.2009, V. Kuznetsova & A. Maryańska-Nadahowska leg.
C. fraudatrix	CFR_f9	f	+	+		POLAND, Bieszczady Mts, Wielka Rawka, 49º06'N; 22º35'E, 07.08.2009, V. Kuznetsova & A. Maryańska-Nadahowska leg.

C. borealis	CBST_f1	f	+	+		FINLAND, Lapland province, Tuntsantie, 67°18'11"N; 29°16'18"E, 01.08.2019, S. Nokkala & Ch. Nokkala leg.
C. borealis	CBST_f3	f	+	+		FINLAND, Lapland province, Tuntsantie, 67°18'11"N; 29°16'18"E, 01.08.2019, S. Nokkala & Ch. Nokkala leg.
C. borealis	CBST_f4	f	+	+		FINLAND, Lapland province, Tuntsantie, 67°18'11"N; 29°16'18"E, 01.08.2019, S. Nokkala & Ch. Nokkala leg.
C. borealis	CBST_f5	f	+	+		FINLAND, Lapland province, Tuntsantie, 67°18'11"N; 29°16'18"E, 01.08.2019, S. Nokkala & Ch. Nokkala leg.
C. borealis	CBST_f6	f	+	+		FINLAND, Lapland province, Tuntsantie, 67°18'11"N; 29°16'18"E, 01.08.2019, S. Nokkala & Ch. Nokkala leg.
C. borealis	CBST_f7	f	-	-		FINLAND, Lapland province, Tuntsantie, 67°18'11"N; 29°16'18"E, 01.08.2019, S. Nokkala & Ch. Nokkala leg.
C. borealis	CBST_f8	f	-	-		FINLAND, Lapland province, Tuntsantie, 67°18'11"N; 29°16'18"E, 01.08.2019, S. Nokkala & Ch. Nokkala leg.
C. borealis	CBST_f9	f	-	-		FINLAND, Lapland province, Tuntsantie, 67°18'11"N; 29°16'18"E, 01.08.2019, S. Nokkala & Ch. Nokkala leg.
C. borealis	CBST_f10	f	-	-		FINLAND, Lapland province, Tuntsantie, 67°18'11"N; 29°16'18"E, 01.08.2019, S. Nokkala & Ch. Nokkala leg.
C. borealis	CBST_f11	f	-	-		FINLAND, Lapland province, Tuntsantie, 67°18'11"N; 29°16'18"E, 01.08.2019, S. Nokkala & Ch. Nokkala leg.
C. borealis	CBST_f12	f	-	-		FINLAND, Lapland province, Tuntsantie, 67°18'11"N; 29°16'18"E, 01.08.2019, S. Nokkala & Ch. Nokkala leg.
C. borealis	CBST_f13	f	-	-		FINLAND, Lapland province, Tuntsantie, 67°18'11"N; 29°16'18"E, 01.08.2019, S. Nokkala & Ch. Nokkala leg.
C. borealis	CBST_f14	f	-	-		FINLAND, Lapland province, Tuntsantie, 67°18'11"N; 29°16'18"E, 01.08.2019, S. Nokkala & Ch. Nokkala leg.
C. borealis	CBST_f15	f	-	-		FINLAND, Lapland province, Tuntsantie, 67°18'11"N; 29°16'18"E, 01.08.2019, S. Nokkala & Ch. Nokkala leg.
C. borealis	CBST_f16	f	-	-		FINLAND, Lapland province, Tuntsantie, 67°18'11"N; 29°16'18"E, 01.08.2019, S. Nokkala & Ch. Nokkala leg.
C. borealis	CBST_f17	f	-	-		FINLAND, Lapland province, Tuntsantie, 67°18'11"N; 29°16'18"E, 01.08.2019, S. Nokkala & Ch. Nokkala leg.
C. borealis	CBST_f18	f	-	-		FINLAND, Lapland province, Tuntsantie, 67°18'11"N; 29°16'18"E, 01.08.2019, S. Nokkala & Ch. Nokkala leg.
C. borealis	CaborPV_f17-1	f	-	-		FINLAND, Lapland province, Inari, Pitkävuono, 68°58'56"N; 26°57'18"E, 16.08.2017, S. Nokkala & Ch. Nokkala leg.
C. borealis	CaborPV_f17-2	f	-	-		FINLAND, Lapland province, Inari, Pitkävuono, 68°58'56"N; 26°57'18"E, 16.08.2017, S. Nokkala & Ch. Nokkala leg.

C. borealis	CaborPV_f17-3	f	-	-		FINLAND, Lapland province, Inari, Pitkävuono, 68°58'56"N; 26°57'18"E, 16.08.2017, S. Nokkala & Ch. Nokkala leg.
C. borealis	CaborPV_f17-4	f	-	-		FINLAND, Lapland province, Inari, Pitkävuono, 68°58'56"N; 26°57'18"E, 16.08.2017, S. Nokkala & Ch. Nokkala leg.
C. borealis	CaborPV_f17-5	f	-	-		FINLAND, Lapland province, Inari, Pitkävuono, 68°58'56"N; 26°57'18"E, 16.08.2017, S. Nokkala & Ch. Nokkala leg.
C. borealis	CBUH_f17-1	f	+	-		FINLAND, Lapland province, Utsjoki, Hietala 69°51'06"N; 27°00'34"E, 15.08.2017, S. Nokkala & Ch. Nokkala leg.
C. borealis	CBUH_f17-2	f	+	-		FINLAND, Lapland province, Utsjoki, Hietala 69°51'06"N; 27°00'34"E, 15.08.2017, S. Nokkala & Ch. Nokkala leg.
C. borealis	CBUH_f17-3	f	-	-		FINLAND, Lapland province, Utsjoki, Hietala 69°51'06"N; 27°00'34"E, 15.08.2012, S. Nokkala & Ch. Nokkala leg.
C. borealis	CBKK_f1	f	-	-		FINLAND, Kuusamo, Kantojoki, 66°14'23"N; 29°09'15"E, 02.08.2019, S. Nokkala & Ch. Nokkala leg.
C. borealis	CBKK_f2	f	-	-		FINLAND, Kuusamo, Kantojoki, 66°14'23"N; 29°09'15"E, 02.08.2019, S. Nokkala & Ch. Nokkala leg.
C. borealis	CBKK_f3	f	-	-		FINLAND, Kuusamo, Kantojoki, 66°14'23"N; 29°09'15"E, 02.08.2019, S. Nokkala & Ch. Nokkala leg.
C. borealis	CBKK_f4	f	-	-		FINLAND, Kuusamo, Kantojoki, 66°14'23"N; 29°09'15"E, 02.08.2019, S. Nokkala & Ch. Nokkala leg.
C. borealis	CBKK_f5	f	-	-		FINLAND, Kuusamo, Kantojoki, 66°14'23"N; 29°09'15"E, 02.08.2019, S. Nokkala & Ch. Nokkala leg.
C. borealis	CBKK_f6	f	-	-		FINLAND, Kuusamo, Kantojoki, 66°14'23"N; 29°09'15"E, 02.08.2019, S. Nokkala & Ch. Nokkala leg.
C. borealis	CBKK_f7	f	-	-		FINLAND, Kuusamo, Kantojoki, 66°14'23"N; 29°09'15"E, 02.08.2019, S. Nokkala & Ch. Nokkala leg.
C. borealis	CBMAG_f1	f	-	-		RUSSIA, Magadan region, vic. Ola vill., 59°34'24"N; 150°46'04"E, 22.07.2020, Yu. Marusik & D.Berman leg.
C. borealis	CBMAG_f2	f	-	-		RUSSIA, Magadan region, vic. Ola vill., 59°34'24"N; 150°46'04"E, 22.07.2020, Yu. Marusik & D.Berman leg.
C. borealis	CBMAG_f3	f	-	-		RUSSIA, Magadan region, vic. Ola vill., 59°34'24"N; 150°46'04"E, 22.07.2020, Yu. Marusik & D.Berman leg.
C. borealis	CBMAG_f4	f	-	-		RUSSIA, Magadan region, vic. Ola vill., 59°34'24"N; 150°46'04"E, 22.07.2020, Yu. Marusik & D.Berman leg.
C. borealis	CBMAG_f5	f	-	-		RUSSIA, Magadan region, vic. Ola vill., 59°34'24"N; 150°46'04"E, 22.07.2020, Yu. Marusik & D.Berman leg.
C. borealis	CBMAG_f6	f	-	-		RUSSIA, Magadan region, vic. Ola vill., 59°34'24"N; 150°46'04"E, 22.07.2020, Yu. Marusik & D.Berman leg.

C. borealis	CBMAG-f7	f	-	-		RUSSIA, Magadan region, vic. Ola vill., 59°34'24"N; 150°46'04"E, 22.07.2020, Yu. Marusik & D.Berman leg.
C. borealis	CBMAG_f8	f	-	-		RUSSIA, Magadan region, vic. Ola vill., 59°34'24"N; 150°46'04"E, 22.07.2020, Yu. Marusik & D.Berman leg.
C. borealis	CBMAG_f9	f	-	-		RUSSIA, Magadan region, vic. Ola vill., 59°34'24"N; 150°46'04"E, 22.07.2020, Yu. Marusik & D.Berman leg.
C. borealis	CBMAG_f10	f	-	-		RUSSIA, Magadan region, vic. Ola vill., 59°34'24"N; 150°46'04"E, 22.07.2020, Yu. Marusik & D.Berman leg.
C. borealis	CBMAG_f11	f	-	-		RUSSIA, Magadan region, vic. Ola vill., 59°34'24"N; 150°46'04"E, 22.07.2020, Yu. Marusik & D.Berman leg.
C. borealis	CBMAG_f12	f	-	-		RUSSIA, Magadan region, vic. Ola vill., 59°34'24"N; 150°46'04"E, 22.07.2020, Yu. Marusik & D.Berman leg.
C. borealis	CV_f1	f	+	+		RUSSIA, Komi Republic, Vorkuta city, 67°27'34"N; 63°59'01"E, 06.08.2013, N. Kabazova & M. Mandelshtam leg.
C. borealis	CV_f11	f	+	+		RUSSIA, Komi Republic, Vorkuta city, 67°27'34"N; 63°59'01"E, 06.08.2013, N. Kabazova & M. Mandelshtam leg.
C. borealis	CBB_f1	f	-	-		RUSSIA, Irkutsk region, Lake Baikal, vic. Bolshye Koty vill. 51°54'25"N, 105°04'14"E, 21.07.2007, E. Labina leg.
C. borealis	CBB_f2	f	-	-		RUSSIA, Irkutsk region, Lake Baikal, vic. Bolshye Koty vill. 51°54'25"N, 105°04'14"E, 21.07.2007, E. Labina leg.
C. borealis	CBB_f3	f	-	-		RUSSIA, Irkutsk region, Lake Baikal, vic. Bolshye Koty vill. 51°54'25"N, 105°04'14"E, 21.07.2007, E. Labina leg.
C. borealis	CBB_f4	f	-	-		RUSSIA, Irkutsk region, Lake Baikal, vic. Bolshye Koty vill. 51°54'25"N, 105°04'14"E, 21.07.2007, E. Labina leg.
C. borealis	CBB_f5	f	-	-		RUSSIA, Irkutsk region, Lake Baikal, vic. Bolshye Koty vill. 51°54'25"N, 105°04'14"E, 21.07.2007, E. Labina leg.
C. borealis	CBB_f6	f	-	-		RUSSIA, Irkutsk region, Lake Baikal, vic. Bolshye Koty vill. 51°54'25"N, 105°04'14"E, 21.07.2007, E. Labina leg.
C. borealis	CLB_f7	f	-	-		RUSSIA, Irkutsk region, Lake Baikal, vic. Bolshye Koty vill. 51°54'25"N, 105°04'14"E, 21.07.2007, E. Labina leg.
C. borealis	CLB_f8	f	-	-		RUSSIA, Irkutsk region, Lake Baikal, vic. Bolshye Koty vill. 51°54'25"N, 105°04'14"E, 21.07.2007, E. Labina leg.
C. borealis	CLB_f9	f	-	-		RUSSIA, Irkutsk region, Lake Baikal, vic. Bolshye Koty vill. 51°54'25"N, 105°04'14"E, 21.07.2007, E. Labina leg.
C. borealis	CLB_f10	f	-	-		RUSSIA, Irkutsk region, Lake Baikal, vic. Bolshye Koty vill. 51°54'25"N, 105°04'14"E, 21.07.2007, E. Labina leg.
C. borealis	CLB_f11	f	-	-		RUSSIA, Irkutsk region, Lake Baikal, vic. Bolshye Koty vill. 51°54'25"N, 105°04'14"E, 21.07.2007, E. Labina leg.

C. borealis	CLB_f12	f	-	-		RUSSIA, Irkutsk region, Lake Baikal, vic. Bolshye Koty vill. 51°54'25"N, 105°04'14"E, 21.07.2007, E. Labina leg.
C. ledi	CLSJ_f51D	f	+	n\a	24+XX	FINLAND, Lapland province, Sevettijärvi, 69°12'58"N; 27°52'14"E, 17.08.2017, S. Nokkala & Ch. Nokkala leg.
C. ledi	CLSJ_f60D	f	+	n\a	24+XX	FINLAND, Lapland province, Sevettijärvi, 69°12'58"N; 27°52'14"E, 17.08.2017, S. Nokkala & Ch. Nokkala leg.
C. ledi	CLSJ_f57T	f	+	n\a	36+XXX	FINLAND, Lapland province, Sevettijärvi, 69°12'58"N; 27°52'14"E, 17.08.2017, S. Nokkala & Ch. Nokkala leg.
C. ledi	CLSJ_f59T	f	+	n\a	36+XXX	FINLAND, Lapland province, Sevettijärvi, 69°12'58"N; 27°52'14"E, 17.08.2017, S. Nokkala & Ch. Nokkala leg.
C. ledi	CLSJ_f61T	f	+	n\a	36+XXX	FINLAND, Lapland province, Sevettijärvi, 69°12'58"N; 27°52'14"E, 17.08.2017, S. Nokkala & Ch. Nokkala leg.
C. ledi	CLSJ_f62T	f	+	n\a	24+XX	FINLAND, Lapland province, Sevettijärvi, 69°12'58"N; 27°52'14"E, 17.08.2017, S. Nokkala & Ch. Nokkala leg.
C. ledi	CacoS1_f19T	f	n\a	+	36+XXX	FINLAND, Lapland province, Sevettijärvi, 69°12'58"N; 27°52'14"E, 17.08.2017, S. Nokkala & Ch. Nokkala leg.
C. ledi	CacoS1_f23T	f	n\a	+	36+XXX	FINLAND, Lapland province, Sevettijärvi, 69°12'58"N; 27°52'14"E, 17.08.2017, S. Nokkala & Ch. Nokkala leg.
C. ledi	CacoS1_f1D	f	n\a	+	24+XX	FINLAND, Lapland province, Sevettijärvi, 69°12'58"N; 27°52'14"E, 17.08.2017, S. Nokkala & Ch. Nokkala leg.
C. ledi	CacoS1_f16D	f	n\a	+	24+XX	FINLAND, Lapland province, Sevettijärvi, 69°12'58"N; 27°52'14"E, 17.08.2017, S. Nokkala & Ch. Nokkala leg.
C. ledi	CacoS1_m2	m	n\a	+	24+X0	FINLAND, Lapland province, Sevettijärvi, 69°12'58"N; 27°52'14"E, 17.08.2017, S. Nokkala & Ch. Nokkala leg.
C. ledi	CacoS1_m3	m	n\a	+	24+X0	FINLAND, Lapland province, Sevettijärvi, 69°12'58"N; 27°52'14"E, 17.08.2017, S. Nokkala & Ch. Nokkala leg.
C. ledi	CLSJ_f1	f	+	n\a		FINLAND, Oulu province, Siikajoki, 64°39'32"N; 25°19'33"E, 10.08.2018, S. Nokkala & Ch. Nokkala leg.
C. ledi	CLSJ_f2	f	+	n\a		FINLAND, Oulu province, Siikajoki, 64°39'32"N; 25°19'33"E, 10.08.2018, S. Nokkala & Ch. Nokkala leg.
C. ledi	CLSJ_f3	f	+	n\a		FINLAND, Oulu province, Siikajoki, 64°39'32"N; 25°19'33"E, 10.08.2018, S. Nokkala & Ch. Nokkala leg.
C. ledi	CLKH_m30	m	+	n\a		FINLAND, Oulu province, Kuhmo, Syväjärvi, 64°11'40"N; 29°57'49"E, 04.08.2019, S. Nokkala & Ch. Nokkala leg.
C. ledi	CLKH_m31	m	+	n\a	24+X0	FINLAND, Oulu province, Kuhmo, Syväjärvi, 64°11'40"N; 29°57'49"E, 04.08.2019, S. Nokkala & Ch. Nokkala leg.
C. ledi	CLKH_m32	m	+	n\a	24+X0	FINLAND, Oulu province, Kuhmo, Syväjärvi, 64°11'40"N; 29°57'49"E, 04.08.2019, S. Nokkala & Ch. Nokkala leg.

C. ledi	CLKH_m33	m	+	n\a		FINLAND, Oulu province, Kuhmo, Syväjärvi, 64°11'40"N; 29°57'49"E, 04.08.2019, S. Nokkala & Ch. Nokkala leg.
C. ledi	CLKH_m34	m	+	n\a		FINLAND, Oulu province, Kuhmo, Syväjärvi, 64°11'40"N; 29°57'49"E, 04.08.2019, S. Nokkala & Ch. Nokkala leg.
C. ledi	CLKH_m35	m	+	n\a		FINLAND, Oulu province, Kuhmo, Syväjärvi, 64°11'40"N; 29°57'49"E, 04.08.2019, S. Nokkala & Ch. Nokkala leg.
C. ledi	CLKH_f1T	f	+	+	36+XXX	FINLAND, Oulu province, Kuhmo, Syväjärvi, 64°11'40"N; 29°57'49"E, 04.08.2019, S. Nokkala & Ch. Nokkala leg.
C. ledi	CLKH_f2D	f	+	+	24+XX	FINLAND, Oulu province, Kuhmo, Syväjärvi, 64°11'40"N; 29°57'49"E, 04.08.2019, S. Nokkala & Ch. Nokkala leg.
C. ledi	CLKH_f3D	f	+	+	24+XX	FINLAND, Oulu province, Kuhmo, Syväjärvi, 64°11'40"N; 29°57'49"E, 04.08.2019, S. Nokkala & Ch. Nokkala leg.
C. ledi	CLKH_f4T	f	+	+	36+XXX	FINLAND, Oulu province, Kuhmo, Syväjärvi, 64°11'40"N; 29°57'49"E, 04.08.2019, S. Nokkala & Ch. Nokkala leg.
C. ledi	CLKH_f5T	f	+	+	36+XXX	FINLAND, Oulu province, Kuhmo, Syväjärvi, 64°11'40"N; 29°57'49"E, 04.08.2019, S. Nokkala & Ch. Nokkala leg.
C. ledi	CLKH_f6T	f	+	+	36+XXX	FINLAND, Oulu province, Kuhmo, Syväjärvi, 64°11'40"N; 29°57'49"E, 04.08.2019, S. Nokkala & Ch. Nokkala leg.
C. ledi	CLYL_m1	m	+		24+X0	FINLAND, Western Finland province, Yläne, 60°53'10"N; 22°26'41"E, 15.08.2019, S. Nokkala & Ch. Nokkala leg.
C. ledi	CLYL_m2	m	+		24+X0	FINLAND, Western Finland province, Yläne, 60°53'10"N; 22°26'41"E, 15.08.2019, S. Nokkala & Ch. Nokkala leg.
C. ledi	CLYL_m3	m	+		24+X0	FINLAND, Western Finland province, Yläne, 60°53'10"N; 22°26'41"E, 15.08.2019, S. Nokkala & Ch. Nokkala leg.
C. ledi	CLYL_m4	m	+		24+X0	FINLAND, Western Finland province, Yläne, 60°53'10"N; 22°26'41"E, 15.08.2019, S. Nokkala & Ch. Nokkala leg.
C. ledi	CLYL_m5	m	+			FINLAND, Western Finland province, Yläne, 60°53'10"N; 22°26'41"E, 15.08.2019, S. Nokkala & Ch. Nokkala leg.
C. ledi	CLYL_m6	m	+			FINLAND, Western Finland province, Yläne, 60°53'10"N; 22°26'41"E, 15.08.2019, S. Nokkala & Ch. Nokkala leg.
C. ledi	CLLMR_m1	m	+		24+X0	FINLAND, Western Finland province, Pöytyä, Lammenrahka, 60°44'37"N; 22°25'32"E, 16.08.2018, S. Nokkala & Ch. Nokkala leg.
C. ledi	CLLMR_m2	m	+		24+X0	FINLAND, Western Finland province, Pöytyä, Lammenrahka, 60°44'37"N; 22°25'32"E, 16.08.2018, S. Nokkala & Ch. Nokkala leg.
C. ledi	CLLMR_m3	m	+		24+X0	FINLAND, Western Finland province, Pöytyä, Lammenrahka, 60°44'37"N; 22°25'32"E, 16.08.2018, S. Nokkala & Ch. Nokkala leg.
C. ledi	CLLMR_m4	m	+		24+X0	FINLAND, Western Finland province, Pöytyä, Lammenrahka, 60°44'37"N; 22°25'32"E, 16.08.2018, S. Nokkala & Ch. Nokkala leg.

C. ledi	CLLMR_m5	m	+			FINLAND, Western Finland province, Pöytyä, Lammenrahka, 60°44'37"N; 22°25'32"E, 16.08.2018, S. Nokkala & Ch. Nokkala leg.
C. ledi	CLLMR_m6	m	+			FINLAND, Western Finland province, Pöytyä, Lammenrahka, 60°44'37"N; 22°25'32"E, 16.08.2018, S. Nokkala & Ch. Nokkala leg.
C. ledi	CLKVI_m1	m	+		24+X0	FINLAND, Western Finland province, Kustavi, 60°39'20"N; 21°18'12"E, 25.08.2019, S. Nokkala & Ch. Nokkala leg.
C. ledi	CLKVI_m2	m	+		24+X0	FINLAND, Western Finland province, Kustavi, 60°39'20"N; 21°18'12"E, 25.08.2019, S. Nokkala & Ch. Nokkala leg.
C. ledi	CLKVI_m3	m	+			FINLAND, Western Finland province, Kustavi, 60°39'20"N; 21°18'12"E, 25.08.2019, S. Nokkala & Ch. Nokkala leg.
C. ledi	CLKVI_m4	m	+			FINLAND, Western Finland province, Kustavi, 60°39'20"N; 21°18'12"E, 25.08.2019, S. Nokkala & Ch. Nokkala leg.
C. ledi	CLKVI_m5	m	+			FINLAND, Western Finland province, Kustavi, 60°39'20"N; 21°18'12"E, 25.08.2019, S. Nokkala & Ch. Nokkala leg.
C. ledi	CLKVI_m6	m	+			FINLAND, Western Finland province, Kustavi, 60°39'20"N; 21°18'12"E, 25.08.2019, S. Nokkala & Ch. Nokkala leg.
C. ledi	PV_17-1	f	+	+		FINLAND, Lapland province, Inari, Pitkävuono, 68°58'56"N; 26°57'18"E, 16.08.2017, S. Nokkala & Ch. Nokkala leg.
C. ledi	CLUH_f1	f	+	+		FINLAND, Lapland province, Utsjoki, Hietala 69°51'06"N; 27°00'34"E, 15.08.2017, S. Nokkala & Ch. Nokkala leg.
C. ledi	CLUH_f2	f	+	+		FINLAND, Lapland province, Utsjoki, Hietala 69°51'06"N; 27°00'34"E, 15.08.2017, S. Nokkala & Ch. Nokkala leg.
C. ledi	CLUH_f3	f	-	+		FINLAND, Lapland province, Utsjoki, Hietala 69°51'06"N; 27°00'34"E, 15.08.2012, S. Nokkala & Ch. Nokkala leg.
C. ledi	CBST_f2	f	+	+		FINLAND, Lapland province, Tuntsantie, 67°18'11"N; 29°16'18"E, 01.08.2019, S. Nokkala & Ch. Nokkala leg.
C. ledi	CLMJ_m1	m	+	+	24+X0	NORWAY, Troms og Finnmark county, Mohkkejogas, 69°26'35"N; 25°11'36"E, 26.07.2020, S. Nokkala & Ch. Nokkala leg.
C. ledi	CLMJ_m2	m	+	+	24+X0	NORWAY, Troms og Finnmark county, Mohkkejogas, 69°26'35"N; 25°11'36"E, 26.07.2020, S. Nokkala & Ch. Nokkala leg.
C. ledi	CLMJ_m3	m	+	-	24+X0	NORWAY, Troms og Finnmark county, Mohkkejogas, 69°26'35"N; 25°11'36"E, 26.07.2020, S. Nokkala & Ch. Nokkala leg.
C. ledi	CLMJ_m4	m	+	+	24+X0	NORWAY, Troms og Finnmark county, Mohkkejogas, 69°26'35"N; 25°11'36"E, 26.07.2020, S. Nokkala & Ch. Nokkala leg.
C. ledi	CLMJ_m5	m	+	+	24+X0	NORWAY, Troms og Finnmark county, Mohkkejogas, 69°26'35"N; 25°11'36"E, 26.07.2020, S. Nokkala & Ch. Nokkala leg.
C. ledi	CLMJ_m6	m	+	+	24+X0	NORWAY, Troms og Finnmark county, Mohkkejogas, 69°26'35"N; 25°11'36"E, 26.07.2020, S. Nokkala & Ch. Nokkala leg.

C. ledi	CLKZ_f1	f	+	-		RUSSIA, Karelia Republic, White Sea, vic. Kolezma vill., 64°14'46"N 35°48'49"E, 30.09.2020, V. Kuznetsova & P. Strelkov leg
C. ledi	CLKZ_f2	f	+	+		RUSSIA, Karelia Republic, White Sea, vic. Kolezma vill., 64°14'46"N 35°48'49"E, 30.09.2020, V. Kuznetsova & P. Strelkov leg
C. ledi	CLKZ_f3	f	+	+		RUSSIA, Karelia Republic, White Sea, vic. Kolezma vill., 64°14'46"N 35°48'49"E, 30.09.2020, V. Kuznetsova & P. Strelkov leg
C. ledi	CLKZ_f4	f	+	+		RUSSIA, Karelia Republic, White Sea, vic. Kolezma vill., 64°14'46"N 35°48'49"E, 30.09.2020, V. Kuznetsova & P. Strelkov leg
C. ledi	CLKX_f5	f	+	+		RUSSIA, Karelia Republic, White Sea, vic. Kolezma vill., 64°14'46"N 35°48'49"E, 30.09.2020, V. Kuznetsova & P. Strelkov leg
C. ledi	CLKZ_f6	f	+	+		RUSSIA, Karelia Republic, White Sea, vic. Kolezma vill., 64°14'46"N 35°48'49"E, 30.09.2020, V. Kuznetsova & P. Strelkov leg
C. ledi	CLKZ_f7	f	+	+		RUSSIA, Karelia Republic, White Sea, vic. Kolezma vill., 64°14'46"N 35°48'49"E, 30.09.2020, V. Kuznetsova & P. Strelkov leg
C. ledi	CLKZ_f8	f	+	-		RUSSIA, Karelia Republic, White Sea, vic. Kolezma vill., 64°14'46"N 35°48'49"E, 30.09.2020, V. Kuznetsova & P. Strelkov leg
C. ledi	CLKZ_f9	f	+	+		RUSSIA, Karelia Republic, White Sea, vic. Kolezma vill., 64°14'46"N 35°48'49"E, 30.09.2020, V. Kuznetsova & P. Strelkov leg
C. ledi	CLKZ_f10	f	+	+		RUSSIA, Karelia Republic, White Sea, vic. Kolezma vill., 64°14'46"N 35°48'49"E, 30.09.2020, V. Kuznetsova & P. Strelkov leg
C. ledi	CLKZ_f11	f	+	+		RUSSIA, Karelia Republic, White Sea, vic. Kolezma vill., 64°14'46"N 35°48'49"E, 30.09.2020, V. Kuznetsova & P. Strelkov leg
C. ledi	CLKZ_f12	f	+	+		RUSSIA, Karelia Republic, White Sea, vic. Kolezma vill., 64°14'46"N 35°48'49"E, 30.09.2020, V. Kuznetsova & P. Strelkov leg
C. ledi	CLKZ_f13	f	+	+		RUSSIA, Karelia Republic, White Sea, vic. Kolezma vill., 64°14'46"N 35°48'49"E, 30.09.2020, V. Kuznetsova & P. Strelkov leg
C. ledi	CLKZ_f14	f	+	+		RUSSIA, Karelia Republic, White Sea, vic. Kolezma vill., 64°14'46"N 35°48'49"E, 30.09.2020, V. Kuznetsova & P. Strelkov leg
C. ledi	CLKZ_f15	f	+	+		RUSSIA, Karelia Republic, White Sea, vic. Kolezma vill., 64°14'46"N 35°48'49"E, 30.09.2020, V. Kuznetsova & P. Strelkov leg
C. ledi	CLKZ_f16	f	+	+		RUSSIA, Karelia Republic, White Sea, vic. Kolezma vill., 64°14'46"N 35°48'49"E, 30.09.2020, V. Kuznetsova & P. Strelkov leg
C. ledi	CLKZ_f17	f	+	+		RUSSIA, Karelia Republic, White Sea, vic. Kolezma vill., 64°14'46"N 35°48'49"E, 30.09.2020, V. Kuznetsova & P. Strelkov leg
C. ledi	CLKZ_f18	f	+	+		RUSSIA, Karelia Republic, White Sea, vic. Kolezma vill., 64°14'46"N 35°48'49"E, 30.09.2020, V. Kuznetsova & P. Strelkov leg
C. ledi	CLV_f2	f	+	+		RUSSIA, Komi Republic, Vorkuta city, 67°27'34"N; 63°59'01"E, 06.08.2013, N. Kabazova & M. Mandelshtam leg.

C. ledi	CLV_f3	f	+	+		RUSSIA, Komi Republic, Vorkuta city, 67°27'34"N; 63°59'01"E, 06.08.2013, N. Kabazova & M. Mandelshtam leg.
C. ledi	CLV_f4	f	+	+		RUSSIA, Komi Republic, Vorkuta city, 67°27'34"N; 63°59'01"E, 06.08.2013, N. Kabazova & M. Mandelshtam leg.
C. ledi	CLV_f5	f	+	+		RUSSIA, Komi Republic, Vorkuta city, 67°27'34"N; 63°59'01"E, 06.08.2013, N. Kabazova & M. Mandelshtam leg.
C. ledi	CLV_f6	f	+	+		RUSSIA, Komi Republic, Vorkuta city, 67°27'34"N; 63°59'01"E, 06.08.2013, N. Kabazova & M. Mandelshtam leg.
C. ledi	CLV_f7	f	+	+		RUSSIA, Komi Republic, Vorkuta city, 67°27'34"N; 63°59'01"E, 06.08.2013, N. Kabazova & M. Mandelshtam leg.
C. ledi	CLV_f8	f	+	+		RUSSIA, Komi Republic, Vorkuta city, 67°27'34"N; 63°59'01"E, 06.08.2013, N. Kabazova & M. Mandelshtam leg.
C. ledi	CLV_f9	f	+	+		RUSSIA, Komi Republic, Vorkuta city, 67°27'34"N; 63°59'01"E, 06.08.2013, N. Kabazova & M. Mandelshtam leg.
C. ledi	CLV_f10	f	+	+		RUSSIA, Komi Republic, Vorkuta city, 67°27'34"N; 63°59'01"E, 06.08.2013, N. Kabazova & M. Mandelshtam leg.
C. ledi	CLV_f12	f	+	+		RUSSIA, Komi Republic, Vorkuta city, 67°27'34"N; 63°59'01"E, 06.08.2013, N. Kabazova & M. Mandelshtam leg.
C. myrtilli	CMPAL_m16	m	+	+	24+X0	FINLAND, Kainuu province, Paltamo district, S of Törmänmäki, 64°33'28"N; 27°43'41"E, 16.07.2009, S. Nokkala & Ch. Nokkala leg.
C. myrtilli	CMPAL_f1	f	+	+		FINLAND, Kainuu province, Paltamo district, S of Törmänmäki, 64°33'28"N; 27°43'41"E, 16.07.2009, S. Nokkala & Ch. Nokkala leg.
C. myrtilli	CMPAL_f2	f	-	-		FINLAND, Kainuu province, Paltamo district, S of Törmänmäki, 64°33'28"N; 27°43'41"E, 16.07.2009, S. Nokkala & Ch. Nokkala leg.
C. myrtilli	CMPAL_f3	f	-	-		FINLAND, Kainuu province, Paltamo district, S of Törmänmäki, 64°33'28"N; 27°43'41"E, 16.07.2009, S. Nokkala & Ch. Nokkala leg.
C. myrtilli	CMPAL_f4	f	-	-		FINLAND, Kainuu province, Paltamo district, S of Törmänmäki, 64°33'28"N; 27°43'41"E, 16.07.2009, S. Nokkala & Ch. Nokkala leg.
C. myrtilli	CMPAL_f5	f	+	+		FINLAND, Kainuu province, Paltamo district, S of Törmänmäki, 64°33'28"N; 27°43'41"E, 16.07.2009, S. Nokkala & Ch. Nokkala leg.
C. myrtilli	CMPAL_f6	f	+	+		FINLAND, Kainuu province, Paltamo district, S of Törmänmäki, 64°33'28"N; 27°43'41"E, 16.07.2009, S. Nokkala & Ch. Nokkala leg.
C. myrtilli	CMPAL_f7	f	-	-		FINLAND, Kainuu province, Paltamo district, S of Törmänmäki, 64°33'28"N; 27°43'41"E, 16.07.2009, S. Nokkala & Ch. Nokkala leg.
C. myrtilli	CMPAL_f8	f	-	-		FINLAND, Kainuu province, Paltamo district, S of Törmänmäki, 64°33'28"N; 27°43'41"E, 16.07.2009, S. Nokkala & Ch. Nokkala leg.
C. myrtilli	CMPAL_f9	f	+	+		FINLAND, Kainuu province, Paltamo district, S of Törmänmäki, 64°33'28"N; 27°43'41"E, 16.07.2009, S. Nokkala & Ch. Nokkala leg.

C. myrtilli	CMPAL_f10	f	+	+		FINLAND, Kainuu province, Paltamo district, S of Törmänmäki, 64°33'28"N; 27°43'41"E, 16.07.2009, S. Nokkala & Ch. Nokkala leg.
C. myrtilli	CMPAL_f11	f	-	-		FINLAND, Kainuu province, Paltamo district, S of Törmänmäki, 64°33'28"N; 27°43'41"E, 16.07.2009, S. Nokkala & Ch. Nokkala leg.
C. myrtilli	CMPAL_f12	f	+	+		FINLAND, Kainuu province, Paltamo district, S of Törmänmäki, 64°33'28"N; 27°43'41"E, 16.07.2009, S. Nokkala & Ch. Nokkala leg.
C. myrtilli	CMUA_m3	m	-	-	24+X0	FINLAND, Lapland province, Utsjoki, Ailigas, 69°53'51"N; 27°03'32"E, 16.08.2017, S. Nokkala & Ch. Nokkala leg.
C. myrtilli	CMUA_f15	f	-	-		FINLAND, Lapland province, Utsjoki, Ailigas, 69°53'51"N; 27°03'32"E, 16.08.2017, S. Nokkala & Ch. Nokkala leg.
C. myrtilli	CMUA_f16	f	+	-		FINLAND, Lapland province, Utsjoki, Ailigas, 69°53'51"N; 27°03'32"E, 16.08.2017, S. Nokkala & Ch. Nokkala leg.
C. myrtilli	CMUA_f17	f	+	-		FINLAND, Lapland province, Utsjoki, Ailigas, 69°53'51"N; 27°03'32"E, 16.08.2017, S. Nokkala & Ch. Nokkala leg.
C. myrtilli	CMUA_f18	f	-	-		FINLAND, Lapland province, Utsjoki, Ailigas, 69°53'51"N; 27°03'32"E, 16.08.2017, S. Nokkala & Ch. Nokkala leg.
C. myrtilli	CMUA_f19	f	-	-		FINLAND, Lapland province, Utsjoki, Ailigas, 69°53'51"N; 27°03'32"E, 16.08.2017, S. Nokkala & Ch. Nokkala leg.
C. myrtilli	CMUA_f20	f	-	-		FINLAND, Lapland province, Utsjoki, Ailigas, 69°53'51"N; 27°03'32"E, 16.08.2017, S. Nokkala & Ch. Nokkala leg.
C. myrtilli	CMUA_f21	f	-	-		FINLAND, Lapland province, Utsjoki, Ailigas, 69°53'51"N; 27°03'32"E, 16.08.2017, S. Nokkala & Ch. Nokkala leg.
C. myrtilli	CMUA_f22	f	-	-		FINLAND, Lapland province, Utsjoki, Ailigas, 69°53'51"N; 27°03'32"E, 16.08.2017, S. Nokkala & Ch. Nokkala leg.
C. myrtilli	CMUA_f23	f	-	-		FINLAND, Lapland province, Utsjoki, Ailigas, 69°53'51"N; 27°03'32"E, 16.08.2017, S. Nokkala & Ch. Nokkala leg.
C. myrtilli	CMUA_f24	f	-	-		FINLAND, Lapland province, Utsjoki, Ailigas, 69°53'51"N; 27°03'32"E, 16.08.2017, S. Nokkala & Ch. Nokkala leg.
C. myrtilli	CMUA_f25	f	-	-		FINLAND, Lapland province, Utsjoki, Ailigas, 69°53'51"N; 27°03'32"E, 16.08.2017, S. Nokkala & Ch. Nokkala leg.
C. myrtilli	CMUA_f26	f	-	-		FINLAND, Lapland province, Utsjoki, Ailigas, 69°53'51"N; 27°03'32"E, 16.08.2017, S. Nokkala & Ch. Nokkala leg.
C. myrtilli	CMUA_f27	f	+	+		FINLAND, Lapland province, Utsjoki, Ailigas, 69°53'51"N; 27°03'32"E, 16.08.2017, S. Nokkala & Ch. Nokkala leg.
C. myrtilli	CMUA_f28	f	-	-		FINLAND, Lapland province, Utsjoki, Ailigas, 69°53'51"N; 27°03'32"E, 16.08.2017, S. Nokkala & Ch. Nokkala leg.
C. myrtilli	CMUH_f7	f	-	-		FINLAND, Lapland province, Utsjoki, Hietala 69°51'06"N; 27°00'34"E, 11.08.2012, S. Nokkala & Ch. Nokkala leg.

C. myrtilli	CMUH_f8	f	-	-		FINLAND, Lapland province, Utsjoki, Hietala 69°51'06"N; 27°00'34"E, 11.08.2012, S. Nokkala & Ch. Nokkala leg.
C. myrtilli	CMUH_f9	f	-	-		FINLAND, Lapland province, Utsjoki, Hietala 69°51'06"N; 27°00'34"E, 11.08.2012, S. Nokkala & Ch. Nokkala leg.
C. myrtilli	CMUH_f10	f	+	+		FINLAND, Lapland province, Utsjoki, Hietala 69°51'06"N; 27°00'34"E, 11.08.2012, S. Nokkala & Ch. Nokkala leg.
C. myrtilli	CMUH_f11	f	-	-		FINLAND, Lapland province, Utsjoki, Hietala 69°51'06"N; 27°00'34"E, 11.08.2012, S. Nokkala & Ch. Nokkala leg.
C. myrtilli	CMUH_f12	f	-	-		FINLAND, Lapland province, Utsjoki, Hietala 69°51'06"N; 27°00'34"E, 11.08.2012, S. Nokkala & Ch. Nokkala leg.
C. myrtilli	CMUH_f13	f	+	-		FINLAND, Lapland province, Utsjoki, Hietala 69°51'06"N; 27°00'34"E, 11.08.2012, S. Nokkala & Ch. Nokkala leg.
C. myrtilli	CMUH_f14	f	+	-		FINLAND, Lapland province, Utsjoki, Hietala 69°51'06"N; 27°00'34"E, 11.08.2012, S. Nokkala & Ch. Nokkala leg.
C. myrtilli	CMUH_f15	f	+	-		FINLAND, Lapland province, Utsjoki, Hietala 69°51'06"N; 27°00'34"E, 11.08.2012, S. Nokkala & Ch. Nokkala leg.
C. myrtilli	CMUH_f16	f	-	-		FINLAND, Lapland province, Utsjoki, Hietala 69°51'06"N; 27°00'34"E, 11.08.2012, S. Nokkala & Ch. Nokkala leg.
C. myrtilli	CMUH_f17	f	+	-		FINLAND, Lapland province, Utsjoki, Hietala 69°51'06"N; 27°00'34"E, 11.08.2012, S. Nokkala & Ch. Nokkala leg.
C. myrtilli	CMUH_f18	f	+	-		FINLAND, Lapland province, Utsjoki, Hietala 69°51'06"N; 27°00'34"E, 11.08.2012, S. Nokkala & Ch. Nokkala leg.
C. myrtilli	CMPS_f7	f	-	n\a		FINLAND, Lapland province, Sodankylä, Puisuvanto, 67°46'52"N; 26°46'09"E, 03.08.2018, S. Nokkala & Ch. Nokkala leg.
C. myrtilli	CMPS_f8	f	-	n\a		FINLAND, Lapland province, Sodankylä, Puisuvanto, 67°46'52"N; 26°46'09"E, 03.08.2018, S. Nokkala & Ch. Nokkala leg.
C. myrtilli	CMPS_f9	f	-	n\a		FINLAND, Lapland province, Sodankylä, Puisuvanto, 67°46'52"N; 26°46'09"E, 03.08.2018, S. Nokkala & Ch. Nokkala leg.
C. myrtilli	CMPS_f10	f	+	n\a		FINLAND, Lapland province, Sodankylä, Puisuvanto, 67°46'52"N; 26°46'09"E, 03.08.2018, S. Nokkala & Ch. Nokkala leg.
C. myrtilli	CMPS_f11	f	+	n\a		FINLAND, Lapland province, Sodankylä, Puisuvanto, 67°46'52"N; 26°46'09"E, 03.08.2018, S. Nokkala & Ch. Nokkala leg.
C. myrtilli	CMPS_f12	f	+	n\a		FINLAND, Lapland province, Sodankylä, Puisuvanto, 67°46'52"N; 26°46'09"E, 03.08.2018, S. Nokkala & Ch. Nokkala leg.
C. myrtilli	CMPS_f13	f	-	n\a		FINLAND, Lapland province, Sodankylä, Puisuvanto, 67°46'52"N; 26°46'09"E, 03.08.2018, S. Nokkala & Ch. Nokkala leg.
C. myrtilli	CMPS_f14	f	-	n\a		FINLAND, Lapland province, Sodankylä, Puisuvanto, 67°46'52"N; 26°46'09"E, 03.08.2018, S. Nokkala & Ch. Nokkala leg.

C. myrtilli	CMPS_f15	f	+	n\a		FINLAND, Lapland province, Sodankylä, Puisuvanto, 67°46'52"N; 26°46'09"E, 03.08.2018, S. Nokkala & Ch. Nokkala leg.
C. myrtilli	CMPS_f16	f	+	n\a		FINLAND, Lapland province, Sodankylä, Puisuvanto, 67°46'52"N; 26°46'09"E, 03.08.2018, S. Nokkala & Ch. Nokkala leg.
C. myrtilli	CMPS_f17	f	+	n\a		FINLAND, Lapland province, Sodankylä, Puisuvanto, 67°46'52"N; 26°46'09"E, 03.08.2018, S. Nokkala & Ch. Nokkala leg.
C. myrtilli	CMPS_f18	f	+	n\a		FINLAND, Lapland province, Sodankylä, Puisuvanto, 67°46'52"N; 26°46'09"E, 03.08.2018, S. Nokkala & Ch. Nokkala leg.
C. myrtilli	CMLI_f17	f	-	-		FINLAND, Olulu province, Liminka, 64°43'53"N; 25°23'04"E, 04.08.2009, S. Nokkala & Ch. Nokkala leg.
C. myrtilli	CMLI_f18	f	-	-		FINLAND, Olulu province, Liminka, 64°43'53"N; 25°23'04"E, 04.08.2009, S. Nokkala & Ch. Nokkala leg.
C. myrtilli	CMLI_f19	f	+	+		FINLAND, Olulu province, Liminka, 64°43'53"N; 25°23'04"E, 04.08.2009, S. Nokkala & Ch. Nokkala leg.
C. myrtilli	CMLI_f20	f	-	-		FINLAND, Olulu province, Liminka, 64°43'53"N; 25°23'04"E, 04.08.2009, S. Nokkala & Ch. Nokkala leg.
C. myrtilli	CMLI_f21	f	+	+		FINLAND, Olulu province, Liminka, 64°43'53"N; 25°23'04"E, 04.08.2009, S. Nokkala & Ch. Nokkala leg.
C. myrtilli	CMLI_f22	f	-	-		FINLAND, Olulu province, Liminka, 64°43'53"N; 25°23'04"E, 04.08.2009, S. Nokkala & Ch. Nokkala leg.
C. myrtilli	CMLI_f23	f	-	-		FINLAND, Olulu province, Liminka, 64°43'53"N; 25°23'04"E, 04.08.2009, S. Nokkala & Ch. Nokkala leg.
C. myrtilli	CMLI_f24	f	-	-		FINLAND, Olulu province, Liminka, 64°43'53"N; 25°23'04"E, 04.08.2009, S. Nokkala & Ch. Nokkala leg.
C. myrtilli	CMLI_f25	f	+	+		FINLAND, Olulu province, Liminka, 64°43'53"N; 25°23'04"E, 04.08.2009, S. Nokkala & Ch. Nokkala leg.
C. myrtilli	CMLI_f26	f	-	-		FINLAND, Olulu province, Liminka, 64°43'53"N; 25°23'04"E, 04.08.2009, S. Nokkala & Ch. Nokkala leg.
C. myrtilli	CMLI_f27	f	+	+		FINLAND, Olulu province, Liminka, 64°43'53"N; 25°23'04"E, 04.08.2009, S. Nokkala & Ch. Nokkala leg.
C. myrtilli	CNLI_f28	f	-	-		FINLAND, Olulu province, Liminka, 64°43'53"N; 25°23'04"E, 04.08.2009, S. Nokkala & Ch. Nokkala leg.
C. myrtilli	CMT_f10	f	+	n\a		FINLAND, Eastern Finland province, Tohmajärvi, 62°23'12"N; 30°19'46"E, 12.08.2012, S. Nokkala & Ch. Nokkala leg.
C. myrtilli	CMT_f11	f	+	n\a		FINLAND, Eastern Finland province, Tohmajärvi, 62°23'12"N; 30°19'46"E, 12.08.2012, S. Nokkala & Ch. Nokkala leg.
C. myrtilli	CMT_f12	f	+	n\a		FINLAND, Eastern Finland province, Tohmajärvi, 62°23'12"N; 30°19'46"E, 12.08.2012, S. Nokkala & Ch. Nokkala leg.

C. myrtilli	CMT_f13	f	+	n\a		FINLAND, Eastern Finland province, Tohmajärvi, 62°23'12"N; 30°19'46"E, 12.08.2012, S. Nokkala & Ch. Nokkala leg.
C. myrtilli	CMT_f14	f	+	n\a		FINLAND, Eastern Finland province, Tohmajärvi, 62°23'12"N; 30°19'46"E, 12.08.2012, S. Nokkala & Ch. Nokkala leg.
C. myrtilli	CMT_f15	f	+	n\a		FINLAND, Eastern Finland province, Tohmajärvi, 62°23'12"N; 30°19'46"E, 12.08.2012, S. Nokkala & Ch. Nokkala leg.
C. myrtilli	CMT_f16	f	+	n\a		FINLAND, Eastern Finland province, Tohmajärvi, 62°23'12"N; 30°19'46"E, 12.08.2012, S. Nokkala & Ch. Nokkala leg.
C. myrtilli	CMT_f17	f	+	n\a		FINLAND, Eastern Finland province, Tohmajärvi, 62°23'12"N; 30°19'46"E, 12.08.2012, S. Nokkala & Ch. Nokkala leg.
C. myrtilli	CMT_f18	f	+	n\a		FINLAND, Eastern Finland province, Tohmajärvi, 62°23'12"N; 30°19'46"E, 12.08.2012, S. Nokkala & Ch. Nokkala leg.
C. myrtilli	CMAB_f1	f	+	+		SWEDEN, Swedish Lapland province, Abisko, Lapporten, 68°19'14"N; 18°51'05"E, h = 570 m, 26.07.2014, S. Nokkala & Ch. Nokkala leg.
C. myrtilli	CMAB_f2	f	+	+		SWEDEN, Swedish Lapland province, Abisko, Lapporten, 68°19'14"N; 18°51'05"E, h = 570 m, 26.07.2014, S. Nokkala & Ch. Nokkala leg.
C. myrtilli	CMAB_f3	f	+	+		SWEDEN, Swedish Lapland province, Abisko, Lapporten, 68°19'14"N; 18°51'05"E, h = 570 m, 26.07.2014, S. Nokkala & Ch. Nokkala leg.
C. myrtilli	CMAB_f4	f	-	-		SWEDEN, Swedish Lapland province, Abisko, Lapporten, 68°19'14"N; 18°51'05"E, h = 570 m, 26.07.2014, S. Nokkala & Ch. Nokkala leg.
C. myrtilli	CMAB_f5	f	+	+		SWEDEN, Swedish Lapland province, Abisko, Lapporten, 68°19'14"N; 18°51'05"E, h = 570 m, 26.07.2014, S. Nokkala & Ch. Nokkala leg.
C. myrtilli	CMAB_f6	f	+	+		SWEDEN, Swedish Lapland province, Abisko, Lapporten, 68°19'14"N; 18°51'05"E, h = 570 m, 26.07.2014, S. Nokkala & Ch. Nokkala leg.
C. myrtilli	CMAB_f7	f	+	+		SWEDEN, Swedish Lapland province, Abisko, Lapporten, 68°19'14"N; 18°51'05"E, h = 570 m, 26.07.2014, S. Nokkala & Ch. Nokkala leg.
C. myrtilli	CMAB_f8	f	+	+		SWEDEN, Swedish Lapland province, Abisko, Lapporten, 68°19'14"N; 18°51'05"E, h = 570 m, 26.07.2014, S. Nokkala & Ch. Nokkala leg.
C. myrtilli	CMAB_f9	f	+	+		SWEDEN, Swedish Lapland province, Abisko, Lapporten, 68°19'14"N; 18°51'05"E, h = 570 m, 26.07.2014, S. Nokkala & Ch. Nokkala leg.
C. myrtilli	CMAB_f10	f	+	-		SWEDEN, Swedish Lapland province, Abisko, Lapporten, 68°19'14"N; 18°51'05"E, h = 570 m, 26.07.2014, S. Nokkala & Ch. Nokkala leg.
C. myrtilli	CMAB_f11	f	+	+		SWEDEN, Swedish Lapland province, Abisko, Lapporten, 68°19'14"N; 18°51'05"E, h = 570 m, 26.07.2014, S. Nokkala & Ch. Nokkala leg.
C. myrtilli	CMAB_f12	f	+	+		SWEDEN, Swedish Lapland province, Abisko, Lapporten, 68°19'14"N; 18°51'05"E, h = 570 m, 26.07.2014, S. Nokkala & Ch. Nokkala leg.
C. myrtilli	CMBF_f13	f	-	-		SWEDEN, Swedish Lapland province, Björkliden, Fjället, 68°24'32"N; 18°39'55"E, 03.08.2009, S. Nokkala & Ch. Nokkala leg.

C. myrtilli	CMBF_f14	f	-	-		SWEDEN, Swedish Lapland province, Björkliden, Fjället, 68°24'32"N; 18°39'55"E, 03.08.2009, S. Nokkala & Ch. Nokkala leg.
C. myrtilli	CMBF_f15	f	-	-		SWEDEN, Swedish Lapland province, Björkliden, Fjället, 68°24'32"N; 18°39'55"E, 03.08.2009, S. Nokkala & Ch. Nokkala leg.
C. myrtilli	CMBF_f16	f	-	-		SWEDEN, Swedish Lapland province, Björkliden, Fjället, 68°24'32"N; 18°39'55"E, 03.08.2009, S. Nokkala & Ch. Nokkala leg.
C. myrtilli	CMBF_f17	f	-	-		SWEDEN, Swedish Lapland province, Björkliden, Fjället, 68°24'32"N; 18°39'55"E, 03.08.2009, S. Nokkala & Ch. Nokkala leg.
C. myrtilli	CMBF_f18	f	-	-		SWEDEN, Swedish Lapland province, Björkliden, Fjället, 68°24'32"N; 18°39'55"E, 03.08.2009, S. Nokkala & Ch. Nokkala leg.
C. myrtilli	CMBF_f19	f	-	-		SWEDEN, Swedish Lapland province, Björkliden, Fjället, 68°24'32"N; 18°39'55"E, 03.08.2009, S. Nokkala & Ch. Nokkala leg.
C. myrtilli	CMBF_f20	f	-	-		SWEDEN, Swedish Lapland province, Björkliden, Fjället, 68°24'32"N; 18°39'55"E, 03.08.2009, S. Nokkala & Ch. Nokkala leg.
C. myrtilli	CMBF_f21	f	-	-		SWEDEN, Swedish Lapland province, Björkliden, Fjället, 68°24'32"N; 18°39'55"E, 03.08.2009, S. Nokkala & Ch. Nokkala leg.
C. myrtilli	CMBF_f22	f	-	-		SWEDEN, Swedish Lapland province, Björkliden, Fjället, 68°24'32"N; 18°39'55"E, 03.08.2009, S. Nokkala & Ch. Nokkala leg.
C. myrtilli	CMBF_f23	f	-	-		SWEDEN, Swedish Lapland province, Björkliden, Fjället, 68°24'32"N; 18°39'55"E, 03.08.2009, S. Nokkala & Ch. Nokkala leg.
C. myrtilli	CMBF_f24	f	-	-		SWEDEN, Swedish Lapland province, Björkliden, Fjället, 68°24'32"N; 18°39'55"E, 03.08.2009, S. Nokkala & Ch. Nokkala leg.
C. myrtilli	CMSO_f7	f	n\a	-		SWEDEN, Norbothnia province, Soppero, 68°00'39"N; 21°39'25"E, 10.08.2012, S. Nokkala & Ch. Nokkala leg.
C. myrtilli	CMSO_f8	f	n\a	-		SWEDEN, Norbothnia province, Soppero, 68°00'39"N; 21°39'25"E, 10.08.2012, S. Nokkala & Ch. Nokkala leg.
C. myrtilli	CMSO_f9	f	n\a	-		SWEDEN, Norbothnia province, Soppero, 68°00'39"N; 21°39'25"E, 10.08.2012, S. Nokkala & Ch. Nokkala leg.
C. myrtilli	CMSO_f10	f	n\a	-		SWEDEN, Norbothnia province, Soppero, 68°00'39"N; 21°39'25"E, 10.08.2012, S. Nokkala & Ch. Nokkala leg.
C. myrtilli	CMSO_f11	f	n\a	-		SWEDEN, Norbothnia province, Soppero, 68°00'39"N; 21°39'25"E, 10.08.2012, S. Nokkala & Ch. Nokkala leg.
C. myrtilli	CMSO_f12	f	n\a	-		SWEDEN, Norbothnia province, Soppero, 68°00'39"N; 21°39'25"E, 10.08.2012, S. Nokkala & Ch. Nokkala leg.
C. myrtilli	CMSO_f16	f	-	-		SWEDEN, Norbothnia province, Soppero, 68°00'39"N; 21°39'25"E, 10.08.2012, S. Nokkala & Ch. Nokkala leg.
C. myrtilli	CMSO_f17	f	-	-		SWEDEN, Norbothnia province, Soppero, 68°00'39"N; 21°39'25"E, 10.08.2012, S. Nokkala & Ch. Nokkala leg.

C. myrtilli	CMSO_f18	f	-	-		SWEDEN, Norbothnia province, Soppero, 68°00'39"N; 21°39'25"E, 10.08.2012, S. Nokkala & Ch. Nokkala leg.
C. myrtilli	CMSO_f19	f	-	-		SWEDEN, Norbothnia province, Soppero, 68°00'39"N; 21°39'25"E, 10.08.2012, S. Nokkala & Ch. Nokkala leg.
C. myrtilli	CMSO_f20	f	-	-		SWEDEN, Norbothnia province, Soppero, 68°00'39"N; 21°39'25"E, 10.08.2012, S. Nokkala & Ch. Nokkala leg.
C. myrtilli	CMSO_f21	f	-	-		SWEDEN, Norbothnia province, Soppero, 68°00'39"N; 21°39'25"E, 10.08.2012, S. Nokkala & Ch. Nokkala leg.
C. myrtilli	CMJO_f9	f	-	n\a		SWEDEN, Norbothnia province, Jokkmok, 66°35'36"N; 19°49'20"E, 08.08.2012, S. Nokkala & Ch. Nokkala leg.
C. myrtilli	CMJO_f10	f	-	n\a		SWEDEN, Norbothnia province, Jokkmok, 66°35'36"N; 19°49'20"E, 08.08.2012, S. Nokkala & Ch. Nokkala leg.
C. myrtilli	CMJO_f11	f	-	n\a		SWEDEN, Norbothnia province, Jokkmok, 66°35'36"N; 19°49'20"E, 08.08.2012, S. Nokkala & Ch. Nokkala leg.
C. myrtilli	CMJO_f12	f	-	n\a		SWEDEN, Norbothnia province, Jokkmok, 66°35'36"N; 19°49'20"E, 08.08.2012, S. Nokkala & Ch. Nokkala leg.
C. myrtilli	CMJO_f13	f	-	n\a		SWEDEN, Norbothnia province, Jokkmok, 66°35'36"N; 19°49'20"E, 08.08.2012, S. Nokkala & Ch. Nokkala leg.
C. myrtilli	CMJO_f14	f	+	n\a		SWEDEN, Norbothnia province, Jokkmok, 66°35'36"N; 19°49'20"E, 08.08.2012, S. Nokkala & Ch. Nokkala leg.
C. myrtilli	CMJO_f21	f	-	-		SWEDEN, Norbothnia province, Jokkmok, 66°35'36"N; 19°49'20"E, 08.08.2012, S. Nokkala & Ch. Nokkala leg.
C. myrtilli	CMJO_f22	f	+	+		SWEDEN, Norbothnia province, Jokkmok, 66°35'36"N; 19°49'20"E, 08.08.2012, S. Nokkala & Ch. Nokkala leg.
C. myrtilli	CMJO_f23	f	-	-		SWEDEN, Norbothnia province, Jokkmok, 66°35'36"N; 19°49'20"E, 08.08.2012, S. Nokkala & Ch. Nokkala leg.
C. myrtilli	CMJO_f24	f	-	-		SWEDEN, Norbothnia province, Jokkmok, 66°35'36"N; 19°49'20"E, 08.08.2012, S. Nokkala & Ch. Nokkala leg.
C. myrtilli	CMJO_f25	f	-	-		SWEDEN, Norbothnia province, Jokkmok, 66°35'36"N; 19°49'20"E, 08.08.2012, S. Nokkala & Ch. Nokkala leg.
C. myrtilli	CMJO_f26	f	-	-		SWEDEN, Norbothnia province, Jokkmok, 66°35'36"N; 19°49'20"E, 08.08.2012, S. Nokkala & Ch. Nokkala leg.
C. myrtilli	CMSS17_f1	f	+	n\a		SWEDEN, Westrobothnia province, Sorsele, 65°29'07"N; 17°33'36"E, 17.08.2010, S. Nokkala & Ch. Nokkala leg.
C. myrtilli	CMSS17_f2	f	+	n\a		SWEDEN, Westrobothnia province, Sorsele, 65°29'07"N; 17°33'36"E, 17.08.2010, S. Nokkala & Ch. Nokkala leg.
C. myrtilli	CMSS17_f3	f	-	n\a		SWEDEN, Westrobothnia province, Sorsele, 65°29'07"N; 17°33'36"E, 17.08.2010, S. Nokkala & Ch. Nokkala leg.

C. myrtilli	CMSS17_f4	f	-	n\a		SWEDEN, Westrobothnia province, Sorsele, 65°29'07"N; 17°33'36"E, 17.08.2010, S. Nokkala & Ch. Nokkala leg.
C. myrtilli	CMSS17_f5	f	-	n\a		SWEDEN, Westrobothnia province, Sorsele, 65°29'07"N; 17°33'36"E, 17.08.2010, S. Nokkala & Ch. Nokkala leg.
C. myrtilli	CMSS17_f6	f	+	n\a		SWEDEN, Westrobothnia province, Sorsele, 65°29'07"N; 17°33'36"E, 17.08.2010, S. Nokkala & Ch. Nokkala leg.
C. myrtilli	CMSS17_f22	f	-	-		SWEDEN, Westrobothnia province, Sorsele, 65°29'07"N; 17°33'36"E, 17.08.2010, S. Nokkala & Ch. Nokkala leg.
C. myrtilli	CMSS17_f23	f	+	+		SWEDEN, Westrobothnia province, Sorsele, 65°29'07"N; 17°33'36"E, 17.08.2010, S. Nokkala & Ch. Nokkala leg.
C. myrtilli	CMSS17_f24	f	+	+		SWEDEN, Westrobothnia province, Sorsele, 65°29'07"N; 17°33'36"E, 17.08.2010, S. Nokkala & Ch. Nokkala leg.
C. myrtilli	CMSS17_f25	f	+	+		SWEDEN, Westrobothnia province, Sorsele, 65°29'07"N; 17°33'36"E, 17.08.2010, S. Nokkala & Ch. Nokkala leg.
C. myrtilli	CMSS17_f26	f	+	+		SWEDEN, Westrobothnia province, Sorsele, 65°29'07"N; 17°33'36"E, 17.08.2010, S. Nokkala & Ch. Nokkala leg.
C. myrtilli	CMSS17_f27	f	-	-		SWEDEN, Westrobothnia province, Sorsele, 65°29'07"N; 17°33'36"E, 17.08.2010, S. Nokkala & Ch. Nokkala leg.
C. myrtilli	CMST_f6	f	n\a	-		SWEDEN, Westrobothnia province, Storuman, 65°05'16"N; 17°06'51"E, 08.08.2012, S. Nokkala & Ch. Nokkala leg.
C. myrtilli	CMST_f7	f	n\a	-		SWEDEN, Westrobothnia province, Storuman, 65°05'16"N; 17°06'51"E, 08.08.2012, S. Nokkala & Ch. Nokkala leg.
C. myrtilli	CMST_f8	f	n\a	+		SWEDEN, Westrobothnia province, Storuman, 65°05'16"N; 17°06'51"E, 08.08.2012, S. Nokkala & Ch. Nokkala leg.
C. myrtilli	CMST_f9	f	n\a	-		SWEDEN, Westrobothnia province, Storuman, 65°05'16"N; 17°06'51"E, 08.08.2012, S. Nokkala & Ch. Nokkala leg.
C. myrtilli	CMST_f10	f	n\a	-		SWEDEN, Westrobothnia province, Storuman, 65°05'16"N; 17°06'51"E, 08.08.2012, S. Nokkala & Ch. Nokkala leg.
C. myrtilli	CMST_f11	f	n\a	+		SWEDEN, Westrobothnia province, Storuman, 65°05'16"N; 17°06'51"E, 08.08.2012, S. Nokkala & Ch. Nokkala leg.
C. myrtilli	CMST_f16	f	-	-		SWEDEN, Westrobothnia province, Storuman, 65°05'16"N; 17°06'51"E, 08.08.2012, S. Nokkala & Ch. Nokkala leg.
C. myrtilli	CMST_f17	f	-	-		SWEDEN, Westrobothnia province, Storuman, 65°05'16"N; 17°06'51"E, 08.08.2012, S. Nokkala & Ch. Nokkala leg.
C. myrtilli	CMST_f18	f	+	+		SWEDEN, Westrobothnia province, Storuman, 65°05'16"N; 17°06'51"E, 08.08.2012, S. Nokkala & Ch. Nokkala leg.
C. myrtilli	CMST_f19	f	-	-		SWEDEN, Westrobothnia province, Storuman, 65°05'16"N; 17°06'51"E, 08.08.2012, S. Nokkala & Ch. Nokkala leg.

C. myrtilli	CMST_f20	f	+	+		SWEDEN, Westrobothnia province, Storuman, 65°05'16"N; 17°06'51"E, 08.08.2012, S. Nokkala & Ch. Nokkala leg.
C. myrtilli	CMST_f21	f	-	-		SWEDEN, Westrobothnia province, Storuman, 65°05'16"N; 17°06'51"E, 08.08.2012, S. Nokkala & Ch. Nokkala leg.
C. myrtilli	CMFIJUO_f7	f	-	-		NORWAY, Troms og Finnmark county, Suoššjavri, 69°22'11"N; 24°18'20"E, 18.08.2011, S. Nokkala & Ch. Nokkala leg.
C. myrtilli	CMFIJUO_f8	f	+	+		NORWAY, Troms og Finnmark county, Suoššjavri, 69°22'11"N; 24°18'20"E, 18.08.2011, S. Nokkala & Ch. Nokkala leg.
C. myrtilli	CMFIJUO_f9	f	-	-		NORWAY, Troms og Finnmark county, Suoššjavri, 69°22'11"N; 24°18'20"E, 18.08.2011, S. Nokkala & Ch. Nokkala leg.
C. myrtilli	CMFIJUO_f10	f	-	-		NORWAY, Troms og Finnmark county, Suoššjavri, 69°22'11"N; 24°18'20"E, 18.08.2011, S. Nokkala & Ch. Nokkala leg.
C. myrtilli	CMFIJUO_f11	f	-	-		NORWAY, Troms og Finnmark county, Suoššjavri, 69°22'11"N; 24°18'20"E, 18.08.2011, S. Nokkala & Ch. Nokkala leg.
C. myrtilli	CMFIJUO_f12	f	-	-		NORWAY, Troms og Finnmark county, Suoššjavri, 69°22'11"N; 24°18'20"E, 18.08.2011, S. Nokkala & Ch. Nokkala leg.
C. myrtilli	CMFI_f13	f	-	-		NORWAY, Troms og Finnmark county, Suoššjavri, 69°22'11"N; 24°18'20"E, 18.08.2011, S. Nokkala & Ch. Nokkala leg.
C. myrtilli	CMFI_f14	f	+	+		NORWAY, Troms og Finnmark county, Suoššjavri, 69°22'11"N; 24°18'20"E, 18.08.2011, S. Nokkala & Ch. Nokkala leg.
C. myrtilli	CMFI_f15	f	+	+		NORWAY, Troms og Finnmark county, Suoššjavri, 69°22'11"N; 24°18'20"E, 18.08.2011, S. Nokkala & Ch. Nokkala leg.
C. myrtilli	CMFI_f16	f	+	-		NORWAY, Troms og Finnmark county, Suoššjavri, 69°22'11"N; 24°18'20"E, 18.08.2011, S. Nokkala & Ch. Nokkala leg.
C. myrtilli	CMFI_f17	f	+	+		NORWAY, Troms og Finnmark county, Suoššjavri, 69°22'11"N; 24°18'20"E, 18.08.2011, S. Nokkala & Ch. Nokkala leg.
C. myrtilli	CMFI_f18	f	-	-		NORWAY, Troms og Finnmark county, Suoššjavri, 69°22'11"N; 24°18'20"E, 18.08.2011, S. Nokkala & Ch. Nokkala leg.
C. myrtilli	CMKLHf09-13	f	-	-		NORWAY, Innlandet county, Sjoa, Kringlothaugen mt., 61°43'06"N; 09°22'40"E, h = 700 m, 01.08.2009, S. Nokkala & Ch. Nokkala leg.
C. myrtilli	CMKLHf09-14	f	-	-		NORWAY, Innlandet county, Sjoa, Kringlothaugen mt., 61°43'06"N; 09°22'40"E, h = 700 m, 01.08.2009, S. Nokkala & Ch. Nokkala leg.
C. myrtilli	CMKLHf09-15	f	-	-		NORWAY, Innlandet county, Sjoa, Kringlothaugen mt., 61°43'06"N; 09°22'40"E, h = 700 m, 01.08.2009, S. Nokkala & Ch. Nokkala leg.
C. myrtilli	CMKLHf09-16	f	-	-		NORWAY, Innlandet county, Sjoa, Kringlothaugen mt., 61°43'06"N; 09°22'40"E, h = 700 m, 01.08.2009, S. Nokkala & Ch. Nokkala leg.
C. myrtilli	CMKLHf09-17	f	-	-		NORWAY, Innlandet county, Sjoa, Kringlothaugen mt., 61°43'06"N; 09°22'40"E, h = 700 m, 01.08.2009, S. Nokkala & Ch. Nokkala leg.

C. myrtilli	CMKLHf09-18	f	-	-		NORWAY, Innlandet county, Sjoa, Kringlothaugen mt., 61°43'06"N; 09°22'40"E, h = 700 m, 01.08.2009, S. Nokkala & Ch. Nokkala leg.
C. myrtilli	CMKV_f09-19	f	-	-		NORWAY, Innlandet county, Sjoa, Kvernbrusaetrin, 61°42'27"N; 09°19'25"E, h = 1100 m, 01.08.2009, S. Nokkala & Ch. Nokkala leg.
C. myrtilli	CMKV_f09-20	f	-	-		NORWAY, Innlandet county, Sjoa, Kvernbrusaetrin, 61°42'27"N; 09°19'25"E, h = 1100 m, 01.08.2009, S. Nokkala & Ch. Nokkala leg.
C. myrtilli	CMKV_f09-21	f	-	-		NORWAY, Innlandet county, Sjoa, Kvernbrusaetrin, 61°42'27"N; 09°19'25"E, h = 1100 m, 01.08.2009, S. Nokkala & Ch. Nokkala leg.
C. myrtilli	CMKV_f09-22	f	-	-		NORWAY, Innlandet county, Sjoa, Kvernbrusaetrin, 61°42'27"N; 09°19'25"E, h = 1100 m, 01.08.2009, S. Nokkala & Ch. Nokkala leg.
C. myrtilli	CMKV_f09-23	f	-	-		NORWAY, Innlandet county, Sjoa, Kvernbrusaetrin, 61°42'27"N; 09°19'25"E, h = 1100 m, 01.08.2009, S. Nokkala & Ch. Nokkala leg.
C. myrtilli	CMKV_f09-24	f	-	-		NORWAY, Innlandet county, Sjoa, Kvernbrusaetrin, 61°42'27"N; 09°19'25"E, h = 1100 m, 01.08.2009, S. Nokkala & Ch. Nokkala leg.
C. myrtilli	CMST_f09-18	f	-	-		NORWAY, Innlandet county, Sjoa, Stålane, 61°41'15"N; 09°14'27"E, h = 1100 m, 01.08.2009, S. Nokkala & Ch. Nokkala leg.
C. myrtilli	CMST_f09-19	f	-	-		NORWAY, Innlandet county, Sjoa, Stålane, 61°41'15"N; 09°14'27"E, h = 1100 m, 01.08.2009, S. Nokkala & Ch. Nokkala leg.
C. myrtilli	CMST_f09-20	f	-	-		NORWAY, Innlandet county, Sjoa, Stålane, 61°41'15"N; 09°14'27"E, h = 1100 m, 01.08.2009, S. Nokkala & Ch. Nokkala leg.
C. myrtilli	CMST_f09-21	f	-	-		NORWAY, Innlandet county, Sjoa, Stålane, 61°41'15"N; 09°14'27"E, h = 1100 m, 01.08.2009, S. Nokkala & Ch. Nokkala leg.
C. myrtilli	CMST_f09-22	f	-	-		NORWAY, Innlandet county, Sjoa, Stålane, 61°41'15"N; 09°14'27"E, h = 1100 m, 01.08.2009, S. Nokkala & Ch. Nokkala leg.
C. myrtilli	CMST_f09-23	f	-	-		NORWAY, Innlandet county, Sjoa, Stålane, 61°41'15"N; 09°14'27"E, h = 1100 m, 01.08.2009, S. Nokkala & Ch. Nokkala leg.
C. myrtilli	CMST_f12-24	f	-	-		NORWAY, Innlandet county, Sjoa, Stålane, 61°41'15"N; 09°14'27"E, h = 1100 m, 01.08.2009, S. Nokkala & Ch. Nokkala leg.
C. myrtilli	CMST_f12-25	f	-	-		NORWAY, Innlandet county, Sjoa, Stålane, 61°41'15"N; 09°14'27"E, h = 1100 m, 01.08.2009, S. Nokkala & Ch. Nokkala leg.
C. myrtilli	CMST_f12-26	f	-	-		NORWAY, Innlandet county, Sjoa, Stålane, 61°41'15"N; 09°14'27"E, h = 1100 m, 01.08.2009, S. Nokkala & Ch. Nokkala leg.
C. myrtilli	CMST_f12-27	f	-	-		NORWAY, Innlandet county, Sjoa, Stålane, 61°41'15"N; 09°14'27"E, h = 1100 m, 01.08.2009, S. Nokkala & Ch. Nokkala leg.
C. myrtilli	CMST_f12-28	f	-	-		NORWAY, Innlandet county, Sjoa, Stålane, 61°41'15"N; 09°14'27"E, h = 1100 m, 01.08.2009, S. Nokkala & Ch. Nokkala leg.
C. myrtilli	CMST_f12-29	f	-	-		NORWAY, Innlandet county, Sjoa, Stålane, 61°41'15"N; 09°14'27"E, h = 1100 m, 01.08.2009, S. Nokkala & Ch. Nokkala leg.

C. myrtilli	CMRU_f26	f	-	-		NORWAY, Innlandet county, Sjoa, Rudihoe, 61°46'27" N; 09°17'16" E, h = 1000 m, 01.08.2009, S. Nokkala & Ch. Nokkala leg.
C. myrtilli	CMRU_f27	f	-	-		NORWAY, Innlandet county, Sjoa, Rudihoe, 61°46'27" N; 09°17'16" E, h = 1000 m, 01.08.2009, S. Nokkala & Ch. Nokkala leg.
C. myrtilli	CMRU_f28	f	-	-		NORWAY, Innlandet county, Sjoa, Rudihoe, 61°46'27" N; 09°17'16" E, h = 1000 m, 01.08.2009, S. Nokkala & Ch. Nokkala leg.
C. myrtilli	CMRU_f29	f	-	-		NORWAY, Innlandet county, Sjoa, Rudihoe, 61°46'27" N; 09°17'16" E, h = 1000 m, 01.08.2009, S. Nokkala & Ch. Nokkala leg.
C. myrtilli	CMRU_f30	f	-	-		NORWAY, Innlandet county, Sjoa, Rudihoe, 61°46'27" N; 09°17'16" E, h = 1000 m, 01.08.2009, S. Nokkala & Ch. Nokkala leg.
C. myrtilli	CMRU_f31	f	-	-		NORWAY, Innlandet county, Sjoa, Rudihoe, 61°46'27" N; 09°17'16" E, h = 1000 m, 01.08.2009, S. Nokkala & Ch. Nokkala leg.
C. myrtilli	CMRU_f32	f	-	-		NORWAY, Innlandet county, Sjoa, Rudihoe, 61°46'27" N; 09°17'16" E, h = 1000 m, 01.08.2009, S. Nokkala & Ch. Nokkala leg.
C. myrtilli	CMRU_f33	f	-	-		NORWAY, Innlandet county, Sjoa, Rudihoe, 61°46'27" N; 09°17'16" E, h = 1000 m, 01.08.2009, S. Nokkala & Ch. Nokkala leg.
C. myrtilli	CMRU_f34	f	-	-		NORWAY, Innlandet county, Sjoa, Rudihoe, 61°46'27" N; 09°17'16" E, h = 1000 m, 01.08.2009, S. Nokkala & Ch. Nokkala leg.
C. myrtilli	CMRU_f35	f	-	-		NORWAY, Innlandet county, Sjoa, Rudihoe, 61°46'27"N; 09°17'16"E, h = 1000 m, 01.08.2009, S. Nokkala & Ch. Nokkala leg.
C. myrtilli	CMRU_f36	f	-	-		NORWAY, Innlandet county, Sjoa, Rudihoe, 61°46'27"N; 09°17'16"E, h = 1000 m, 01.08.2009, S. Nokkala & Ch. Nokkala leg.
C. myrtilli	CMRU_f37	f	-	-		NORWAY, Innlandet county, Sjoa, Rudihoe, 61°46'27"N; 09°17'16"E, h = 1000 m, 01.08.2009, S. Nokkala & Ch. Nokkala leg.
C. myrtilli	CMRI_f14	f	-	-		NORWAY, Innlandet county, Sjoa, Rindhovda, 61°43'05"N; 09°05'12"E, h = 1080 m, 16.08.2010, S. Nokkala & Ch. Nokkala leg.
C. myrtilli	CMRI_f15	f	-	-		NORWAY, Innlandet county, Sjoa, Rindhovda, 61°43'05"N; 09°05'12"E, h = 1080 m, 16.08.2010, S. Nokkala & Ch. Nokkala leg.
C. myrtilli	CMRI_f16	f	-	-		NORWAY, Innlandet county, Sjoa, Rindhovda, 61°43'05"N; 09°05'12"E, h = 1080 m, 16.08.2010, S. Nokkala & Ch. Nokkala leg.
C. myrtilli	CMRI_f17	f	-	-		NORWAY, Innlandet county, Sjoa, Rindhovda, 61°43'05"N; 09°05'12"E, h = 1080 m, 16.08.2010, S. Nokkala & Ch. Nokkala leg.
C. myrtilli	CMRI_f18	f	-	-		NORWAY, Innlandet county, Sjoa, Rindhovda, 61°43'05"N; 09°05'12"E, h = 1080 m, 16.08.2010, S. Nokkala & Ch. Nokkala leg.
C. myrtilli	CMRI_f19	f	-	-		NORWAY, Innlandet county, Sjoa, Rindhovda, 61°43'05"N; 09°05'12"E, h = 1080 m, 16.08.2010, S. Nokkala & Ch. Nokkala leg.
C. myrtilli	CMRI_f20	f	-	-		NORWAY, Innlandet county, Sjoa, Rindhovda, 61°43'05"N; 09°05'12"E, h = 1080 m, 16.08.2010, S. Nokkala & Ch. Nokkala leg.

C. myrtilli	CMRI_f21	f	-	-		NORWAY, Innlandet county, Sjoa, Rindhovda, 61°43'05"N; 09°05'12"E, h = 1080 m, 16.08.2010, S. Nokkala & Ch. Nokkala leg.
C. myrtilli	CMRI_f22	f	-	-		NORWAY, Innlandet county, Sjoa, Rindhovda, 61°43'05"N; 09°05'12"E, h = 1080 m, 16.08.2010, S. Nokkala & Ch. Nokkala leg.
C. myrtilli	CMRI_f23	f	-	-		NORWAY, Innlandet county, Sjoa, Rindhovda, 61°43'05"N; 09°05'12"E, h = 1080 m, 16.08.2010, S. Nokkala & Ch. Nokkala leg.
C. myrtilli	CMRI_f24	f	-	-		NORWAY, Innlandet county, Sjoa, Rindhovda, 61°43'05"N; 09°05'12"E, h = 1080 m, 16.08.2010, S. Nokkala & Ch. Nokkala leg.
C. myrtilli	CMKILD_f1	f	-	-		RUSSIA, Murmansk region, Barents Sea, Kildin Island, 69°19'58"N; 34°23'43"E, 05.08.2016, P. Strelkov leg.
C. myrtilli	CMKILD_f2	f	-	-		RUSSIA, Murmansk region, Barents Sea, Kildin Island, 69°19'58"N; 34°23'43"E, 05.08.2016, P. Strelkov leg.
C. myrtilli	CMKILD_f3	f	-	-		RUSSIA, Murmansk region, Barents Sea, Kildin Island, 69°19'58"N; 34°23'43"E, 05.08.2016, P. Strelkov leg.
C. myrtilli	CMKILD_f4	f	-	-		RUSSIA, Murmansk region, Barents Sea, Kildin Island, 69°19'58"N; 34°23'43"E, 05.08.2016, P. Strelkov leg.
C. myrtilli	CMKILD_f5	f	-	-		RUSSIA, Murmansk region, Barents Sea, Kildin Island, 69°19'58"N; 34°23'43"E, 05.08.2016, P. Strelkov leg.
C. myrtilli	CMKILD_f6	f	-	-		RUSSIA, Murmansk region, Barents Sea, Kildin Island, 69°19'58"N; 34°23'43"E, 05.08.2016, P. Strelkov leg.
C. myrtilli	CMKILD_f7	f	-	-		RUSSIA, Murmansk region, Barents Sea, Kildin Island, 69°19'58"N; 34°23'43"E, 05.08.2016, P. Strelkov leg.
C. myrtilli	CMKILD_f8	f	-	-		RUSSIA, Murmansk region, Barents Sea, Kildin Island, 69°19'58"N; 34°23'43"E, 05.08.2016, P. Strelkov leg.
C. myrtilli	CMKILD_f9	f	-	-		RUSSIA, Murmansk region, Barents Sea, Kildin Island, 69°19'58"N; 34°23'43"E, 05.08.2016, P. Strelkov leg.
C. myrtilli	CMKILD_f10	f	-	-		RUSSIA, Murmansk region, Barents Sea, Kildin Island, 69°19'58"N; 34°23'43"E, 05.08.2016, P. Strelkov leg.
C. myrtilli	CMKILD_f11	f	-	-		RUSSIA, Murmansk region, Barents Sea, Kildin Island, 69°19'58"N; 34°23'43"E, 05.08.2016, P. Strelkov leg.
C. myrtilli	CMKILD_f12	f	-	-		RUSSIA, Murmansk region, Barents Sea, Kildin Island, 69°19'58"N; 34°23'43"E, 05.08.2016, P. Strelkov leg.
C. myrtilli	CMKILD_f13	f	n\a	-		RUSSIA, Murmansk region, Barents Sea, Kildin Island, 69°19'58"N; 34°23'43"E, 05.08.2016, P. Strelkov leg.
C. myrtilli	CMKILD_f14	f	n\a	-		RUSSIA, Murmansk region, Barents Sea, Kildin Island, 69°19'58"N; 34°23'43"E, 05.08.2016, P. Strelkov leg.
C. myrtilli	CMKILD_f25	f	n\a	-		RUSSIA, Murmansk region, Barents Sea, Kildin Island, 69°19'58"N; 34°23'43"E, 05.08.2016, P. Strelkov leg.

C. myrtilli	CMKILD_f26	f	n\a	-		RUSSIA, Murmansk region, Barents Sea, Kildin Island, 69°19'58"N; 34°23'43"E, 05.08.2016, P. Strelkov leg.
C. myrtilli	CMKILD_f27	f	n\a	-		RUSSIA, Murmansk region, Barents Sea, Kildin Island, 69°19'58"N; 34°23'43"E, 05.08.2016, P. Strelkov leg.
C. myrtilli	CMKILD_f28	f	n\a	-		RUSSIA, Murmansk region, Barents Sea, Kildin Island, 69°19'58"N; 34°23'43"E, 05.08.2016, P. Strelkov leg.
C. myrtilli	CM4.1	m	-	-		RUSSIA, Murmansk region, Laplandsky Natural Reserve, 68°07'N; 32°27'E, 01.08.2019, A. Polevoi leg.
C. myrtilli	CM4.2	m	-	-		RUSSIA, Murmansk region, Laplandsky Natural Reserve, 68°07'N; 32°27'E, 01.08.2019, A. Polevoi leg.
C. myrtilli	CM7.1	m	-	-		RUSSIA, Murmansk region, Laplandsky Natural Reserve, 68°07'N; 32°27'E, 01.08.2019, A. Polevoi leg.
C. myrtilli	CM7.2	f	-	-		RUSSIA, Murmansk region, Laplandsky Natural Reserve, 68°07'N; 32°27'E, 01.08.2019, A. Polevoi leg.
C. myrtilli	CM7.3	f	+	+		RUSSIA, Murmansk region, Laplandsky Natural Reserve, 68°07'N; 32°27'E, 01.08.2019, A. Polevoi leg.
C. myrtilli	CM7.4	f	+	+		RUSSIA, Murmansk region, Laplandsky Natural Reserve, 68°07'N; 32°27'E, 01.08.2019, A. Polevoi leg.
C. myrtilli	CM7.5	f	-	-		RUSSIA, Murmansk region, Laplandsky Natural Reserve, 68°07'N; 32°27'E, 01.08.2019, A. Polevoi leg.
C. myrtilli	CM7.6	f	+	+		RUSSIA, Murmansk region, Laplandsky Natural Reserve, 68°07'N; 32°27'E, 01.08.2019, A. Polevoi leg.
C. myrtilli	CM7.7	f	+	+		RUSSIA, Murmansk region, Laplandsky Natural Reserve, 68°07'N; 32°27'E, 01.08.2019, A. Polevoi leg.
C. myrtilli	CM7.8	f	-	-		RUSSIA, Murmansk region, Laplandsky Natural Reserve, 68°07'N; 32°27'E, 01.08.2019, A. Polevoi leg.
C. myrtilli	CM7.9	f	+	+		RUSSIA, Murmansk region, Laplandsky Natural Reserve, 68°07'N; 32°27'E, 01.08.2019, A. Polevoi leg.
C. myrtilli	CM7.10	f	-	-		RUSSIA, Murmansk region, Laplandsky Natural Reserve, 68°07'N; 32°27'E, 01.08.2019, A. Polevoi leg.
C. myrtilli	CM7.11	f	+	+		RUSSIA, Murmansk region, Laplandsky Natural Reserve, 68°07'N; 32°27'E, 01.08.2019, A. Polevoi leg.
C. myrtili	CMKOL_f1	f	-	n\a		RUSSIA, Karelia Republic, White Sea, vic. Kolezma vill., 64°14'46"N 35°48'49"E, 30.09.2020, V. Kuznetsova & P. Strelkov leg.
C. myrtilli	CM5.1	f	-	-		RUSSIA, Karelia Republic, Louchskiy district, "Belomorskaya" Research Station, 66°17'58"N; 33°37'18"E, 29.08.2019, G. Paskerova leg.
C. myrtilli	CM5.2	f	+	+		RUSSIA, Karelia Republic, Louchskiy district, "Belomorskaya" Research Station, 66°17'58"N; 33°37'18"E, 29.08.2019, G. Paskerova leg.

C. myrtilli	CM5.3	f	-	-		RUSSIA, Karelia Republic, Louchskiy district, “Belomorskaya” Research Station, 66°17'58"N; 33°37'18"E, 29.08.2019, G. Paskerova leg.
C. myrtilli	CM5.4	f	+	+		RUSSIA, Karelia Republic, Louchskiy district, “Belomorskaya” Research Station, 66°17'58"N; 33°37'18"E, 29.08.2019, G. Paskerova leg.
C. myrtilli	CM5.5	f	-	-		RUSSIA, Karelia Republic, Louchskiy district, “Belomorskaya” Research Station, 66°17'58"N; 33°37'18"E, 29.08.2019, G. Paskerova leg.
C. myrtilli	CM5.6	f	-	-		RUSSIA, Karelia Republic, Louchskiy district, “Belomorskaya” Research Station, 66°17'58"N; 33°37'18"E, 29.08.2019, G. Paskerova leg.
C. myrtilli	CM5.7	f	-	-		RUSSIA, Karelia Republic, Louchskiy district, “Belomorskaya” Research Station, 66°17'58"N; 33°37'18"E, 29.08.2019, G. Paskerova leg.
C. myrtilli	CM5.8	f	-	-		RUSSIA, Karelia Republic, Louchskiy district, “Belomorskaya” Research Station, 66°17'58"N; 33°37'18"E, 29.08.2019, G. Paskerova leg.
C. myrtilli	CM5.9	f	-	-		RUSSIA, Karelia Republic, Louchskiy district, “Belomorskaya” Research Station, 66°17'58"N; 33°37'18"E, 29.08.2019, G. Paskerova leg.
C. myrtilli	CM5.10	f	-	-		RUSSIA, Karelia Republic, Louchskiy district, “Belomorskaya” Research Station, 66°17'58"N; 33°37'18"E, 29.08.2019, G. Paskerova leg.
C. myrtilli	CM1.1	f	-	-		RUSSIA, Karelia Republic, White Sea, Sredniy Island, 66°17'28"N; 33°39'06"E, 21.08.2017, G. Paskerova leg.
C. myrtilli	CM1.2	f	+	+		RUSSIA, Karelia Republic, White Sea, Sredniy Island, 66°17'28"N; 33°39'06"E, 21.08.2017, G. Paskerova leg.
C. myrtilli	CM1.3	f	+	+		RUSSIA, Karelia Republic, White Sea, Sredniy Island, 66°17'28"N; 33°39'06"E, 21.08.2017, G. Paskerova leg.
C. myrtilli	CM1.4	f	-	-		RUSSIA, Karelia Republic, White Sea, Sredniy Island, 66°17'28"N; 33°39'06"E, 21.08.2017, G. Paskerova leg.
C. myrtilli	CM1.5	f	-	-		RUSSIA, Karelia Republic, White Sea, Sredniy Island, 66°17'28"N; 33°39'06"E, 21.08.2017, G. Paskerova leg.
C. myrtilli	CM1.6	f	-	-		RUSSIA, Karelia Republic, White Sea, Sredniy Island, 66°17'28"N; 33°39'06"E, 21.08.2017, G. Paskerova leg.
C. myrtilli	CM1.7	f	-	-		RUSSIA, Karelia Republic, White Sea, Sredniy Island, 66°17'28"N; 33°39'06"E, 21.08.2017, G. Paskerova leg.
C. myrtilli	CM1a.1	f	-	-		RUSSIA, Karelia Republic, White Sea, Sredniy Island, 66°17'28"N; 33°39'06"E, 21.08.2017, G. Paskerova leg.
C. myrtilli	CM1a.2	f	+	+		RUSSIA, Karelia Republic, White Sea, Sredniy Island, 66°17'28"N; 33°39'06"E, 21.08.2017, G. Paskerova leg.
C. myrtilli	CM1a.3	f	+	+		RUSSIA, Karelia Republic, White Sea, Sredniy Island, 66°17'28"N; 33°39'06"E, 21.08.2017, G. Paskerova leg.
C. myrtilli	CM1a.4	f	-	-		RUSSIA, Karelia Republic, White Sea, Sredniy Island, 66°17'28"N; 33°39'06"E, 21.08.2017, G. Paskerova leg.

C. myrtilli	CM1a.5	f	+	+		RUSSIA, Karelia Republic, White Sea, Sredniy Island, 66°17'28"N; 33°39'06"E, 21.08.2017, G. Paskerova leg.
C. myrtilli	CM2.1	f	+	+		RUSSIA, Karelia Republic, White Sea, Sredniy Island, 66°17'28"N; 33°39'06"E, 21.08.2017, G. Paskerova leg.
C. myrtilli	CM2.2	f	-	-		RUSSIA, Karelia Republic, White Sea, Sredniy Island, 66°17'28"N; 33°39'06"E, 21.08.2017, G. Paskerova leg.
C. myrtilli	CM2.3	f	-	-		RUSSIA, Karelia Republic, White Sea, Sredniy Island, 66°17'28"N; 33°39'06"E, 21.08.2017, G. Paskerova leg.
C. myrtilli	CM2.4	f	-	-		RUSSIA, Karelia Republic, White Sea, Sredniy Island, 66°17'28"N; 33°39'06"E, 21.08.2017, G. Paskerova leg.
C. myrtilli	CM2.5	f	-	-		RUSSIA, Karelia Republic, White Sea, Sredniy Island, 66°17'28"N; 33°39'06"E, 21.08.2017, G. Paskerova leg.
C. myrtilli	CM3.1	f	-	-		RUSSIA, Karelia Republic, vic. Kem’ city, 2017.
C. myrtilli	CM3.2	f	+	+		RUSSIA, Karelia Republic, vic. Kem’ city, 2017.
C. myrtilli	CM3.3	f	-	-		RUSSIA, Karelia Republic, vic. Kem’ city, 2017.
C. myrtilli	CM3.4	f	-	-		RUSSIA, Karelia Republic, vic. Kem’ city, 2017.
C. myrtilli	CM3.5	f	-	-		RUSSIA, Karelia Republic, vic. Kem’ city, 2017.
C. myrtilli	CM3.6	f	+	+		RUSSIA, Karelia Republic, vic. Kem’ city, 2017.
C. myrtilli	CM3.7	f	-	-		RUSSIA, Karelia Republic, vic. Kem’ city, 2017.
C. myrtilli	CM3.8	f	-	-		RUSSIA, Karelia Republic, vic. Kem’ city, 2017.
C. myrtilli	CM3.9	f	-	-		RUSSIA, Karelia Republic, vic. Kem’ city, 2017.
C. myrtilli	CM3.10	f	-	-		RUSSIA, Karelia Republic, vic. Kem’ city, 2017.
C. myrtilli	CM3.11	f	-	-		RUSSIA, Karelia Republic, vic. Kem’ city, 2017.
C. myrtilli	CM3.12	f	-	-		RUSSIA, Karelia Republic, vic. Kem’ city, 2017.
C. myrtilli	CMMAG_f1	f	n\a	-		RUSSIA, Magadan region, vic. Ola vill., 59°34'24"N; 150°46'04"E, 22.07.2020, Yu. Marusik & D. Berman leg.
C. myrtilli	CMMAG_f2	f	n\a	-		RUSSIA, Magadan region, vic. Ola vill., 59°34'24"N; 150°46'04"E, 22.07.2020, Yu. Marusik & D. Berman leg.
C. myrtilli	CMMAG_f3	f	n\a	-		RUSSIA, Magadan region, vic. Ola vill., 59°34'24"N; 150°46'04"E, 22.07.2020, Yu. Marusik & D. Berman leg.
C. myrtilli	CMMAG_f4	f	n\a	-		RUSSIA, Magadan region, vic. Ola vill., 59°34'24"N; 150°46'04"E, 22.07.2020, Yu. Marusik & D. Berman leg.
C. myrtilli	CMMAG_f5	f	n\a	-		RUSSIA, Magadan region, vic. Ola vill., 59°34'24"N; 150°46'04"E, 22.07.2020, Yu. Marusik & D. Berman leg.
C. myrtilli	CMMAG_f6	f	n\a	-		RUSSIA, Magadan region, vic. Ola vill., 59°34'24"N; 150°46'04"E, 22.07.2020, Yu. Marusik & D. Berman leg.

C. myrtilli	CMMAG_f7	f	n\a	-		RUSSIA, Magadan region, vic. Ola vill., 59°34'24"N; 150°46'04"E, 22.07.2020, Yu. Marusik & D. Berman leg.
C. myrtilli	CMMAG_f8	f	n\a	-		RUSSIA, Magadan region, vic. Ola vill., 59°34'24"N; 150°46'04"E, 22.07.2020, Yu. Marusik & D. Berman leg.
C. myrtilli	CMMAG_f9	f	n\a	-		RUSSIA, Magadan region, vic. Ola vill., 59°34'24"N; 150°46'04"E, 22.07.2020, Yu. Marusik & D. Berman leg.
C. myrtilli	CMMAG_f10	f	n\a	+		RUSSIA, Magadan region, vic. Ola vill., 59°34'24"N; 150°46'04"E, 22.07.2020, Yu. Marusik & D. Berman leg.
C. myrtilli	CMMAG_f11	f	n\a	-		RUSSIA, Magadan region, vic. Ola vill., 59°34'24"N; 150°46'04"E, 22.07.2020, Yu. Marusik & D. Berman leg.
C. myrtilli	CMMAG_f12	f	n\a	-		RUSSIA, Magadan region, vic. Ola vill., 59°34'24"N; 150°46'04"E, 22.07.2020, Yu. Marusik & D. Berman leg.
C. myrtilli	CMMAG_f13	f	n\a	-		RUSSIA, Magadan region, vic. Ola vill., 59°34'24"N; 150°46'04"E, 22.07.2020, Yu. Marusik & D. Berman leg.
C. myrtilli	CMMAG_f14	f	n\a	-		RUSSIA, Magadan region, vic. Ola vill., 59°34'24"N; 150°46'04"E, 22.07.2020, Yu. Marusik & D. Berman leg.
C. myrtilli	CMMAG_f15	f	n\a	-		RUSSIA, Magadan region, vic. Ola vill., 59°34'24"N; 150°46'04"E, 22.07.2020, Yu. Marusik & D. Berman leg.
C. myrtilli	CMSYK_f1	f	-	-		RUSSIA, Komi Republic, 2km W of Syktyvkar city, 61°38'57"N; 50°44'09"E, 02.09.2020, A. Zinovieva leg.
C. myrtilli	CMSYK_f2	f	-	-		RUSSIA, Komi Republic, 2km W of Syktyvkar city, 61°38'57"N; 50°44'09"E, 02.09.2020, A. Zinovieva leg.
C. myrtilli	CMSYK_f3	f	-	-		RUSSIA, Komi Republic, 2km W of Syktyvkar city, 61°38'57"N; 50°44'09"E, 02.09.2020, A. Zinovieva leg.
C. myrtilli	CMSYK_f4	f	-	-		RUSSIA, Komi Republic, 2km W of Syktyvkar city, 61°38'57"N; 50°44'09"E, 02.09.2020, A. Zinovieva leg.
C. myrtilli	CMSYK_f5	f	-	-		RUSSIA, Komi Republic, 2km W of Syktyvkar city, 61°38'57"N; 50°44'09"E, 02.09.2020, A. Zinovieva leg.
C. myrtilli	CMSYK_f6	f	-	-		RUSSIA, Komi Republic, 2km W of Syktyvkar city, 61°38'57"N; 50°44'09"E, 02.09.2020, A. Zinovieva leg.
C. myrtilli	CMSYK_f7	f	-	-		RUSSIA, Komi Republic, 2km W of Syktyvkar city, 61°38'57"N; 50°44'09"E, 02.09.2020, A. Zinovieva leg.
C. myrtilli	CMSYK_f8	f	-	-		RUSSIA, Komi Republic, 2km W of Syktyvkar city, 61°38'57"N; 50°44'09"E, 02.09.2020, A. Zinovieva leg.
C. myrtilli	CMSYK_f9	f	-	-		RUSSIA, Komi Republic, 2km W of Syktyvkar city, 61°38'57"N; 50°44'09"E, 02.09.2020, A. Zinovieva leg.
C. myrtilli	CMSYK_f10	f	-	-		RUSSIA, Komi Republic, 2km W of Syktyvkar city, 61°38'57"N; 50°44'09"E, 02.09.2020, A. Zinovieva leg.

C. myrtilli	CMSYK_f11	f	-	-		RUSSIA, Komi Republic, 2km W of Syktyvkar city, 61°38'57"N; 50°44'09"E, 02.09.2020, A. Zinovieva leg.
C. myrtilli	CMSYK_f12	f	-	-		RUSSIA, Komi Republic, 2km W of Syktyvkar city, 61°38'57"N; 50°44'09"E, 02.09.2020, A. Zinovieva leg.
C. myrtilli	CMSYK_f13	f	-	-		RUSSIA, Komi Republic, 2km W of Syktyvkar city, 61°38'57"N; 50°44'09"E, 02.09.2020, A. Zinovieva leg.
C. myrtilli	CMV_f14	f	-	-		RUSSIA, Komi Republic, Vorkuta city, 67°27'34"N; 63°59'01"E, 06.08.2013, N. Kabazova & M. Mandelshtam leg.
C. myrtilli	CMV_f15	f	-	-		RUSSIA, Komi Republic, Vorkuta city, 67°27'34"N; 63°59'01"E, 06.08.2013, N. Kabazova & M. Mandelshtam leg.
C. myrtilli	CMV_f16	f	-	-		RUSSIA, Komi Republic, Vorkuta city, 67°27'34"N; 63°59'01"E, 06.08.2013, N. Kabazova & M. Mandelshtam leg.
C. myrtilli	CMV_f17	f	-	-		RUSSIA, Komi Republic, Vorkuta city, 67°27'34"N; 63°59'01"E, 06.08.2013, N. Kabazova & M. Mandelshtam leg.
C. myrtilli	CMV_f18	f	-	-		RUSSIA, Komi Republic, Vorkuta city, 67°27'34"N; 63°59'01"E, 06.08.2013, N. Kabazova & M. Mandelshtam leg.
C. myrtilli	CMV_f19	f	-	-		RUSSIA, Komi Republic, Vorkuta city, 67°27'34"N; 63°59'01"E, 06.08.2013, N. Kabazova & M. Mandelshtam leg.
C. myrtilli	CMV_f20	f	-	-		RUSSIA, Komi Republic, Vorkuta city, 67°27'34"N; 63°59'01"E, 06.08.2013, N. Kabazova & M. Mandelshtam leg.
C. myrtilli	CMV_f21	f	-	-		RUSSIA, Komi Republic, Vorkuta city, 67°27'34"N; 63°59'01"E, 06.08.2013, N. Kabazova & M. Mandelshtam leg.
C. myrtilli	CMV_f22	f	+	+		RUSSIA, Komi Republic, Vorkuta city, 67°27'34"N; 63°59'01"E, 06.08.2013, N. Kabazova & M. Mandelshtam leg.
C. myrtilli	CMV_f23	f	-	-		RUSSIA, Komi Republic, Vorkuta city, 67°27'34"N; 63°59'01"E, 06.08.2013, N. Kabazova & M. Mandelshtam leg.
C. myrtilli	CMV_f24	f	-	-		RUSSIA, Komi Republic, Vorkuta city, 67°27'34"N; 63°59'01"E, 06.08.2013, N. Kabazova & M. Mandelshtam leg.
C. myrtilli	CMV_f25	f	-	-		RUSSIA, Komi Republic, Vorkuta city, 67°27'34"N; 63°59'01"E, 06.08.2013, N. Kabazova & M. Mandelshtam leg.
C. myrtilli	CMKEM_f5.1	f	-	-		RUSSIA, Kemerovo region, Kemerovskiy district, vic. Voskresenka vill., 55°19'N; 86°48'E, July 2019. A. Polevoi leg.
C. myrtilli	CMKEM_f5.2	f	-	-		RUSSIA, Kemerovo region, Kemerovskiy district, vic. Voskresenka vill., 55°19'N; 86°48'E, July 2019. A. Polevoi leg.
C. myrtilli	CMKEM_f5.3	f	-	-		RUSSIA, Kemerovo region, Kemerovskiy district, vic. Voskresenka vill., 55°19'N; 86°48'E, July 2019. A. Polevoi leg.
C. myrtilli	CMKEM_f5.4	f	-	-		RUSSIA, Kemerovo region, Kemerovskiy district, vic. Voskresenka vill., 55°19'N; 86°48'E, July 2019. A. Polevoi leg.

C. myrtilli	CMKEM_f5.5	f	-	-		RUSSIA, Kemerovo region, Kemerovskiy district, vic. Voskresenka vill., 55°19'N; 86°48'E, July 2019. A. Polevoi leg.
C. myrtilli	CMKEM_f5.6	f	-	-		RUSSIA, Kemerovo region, Kemerovskiy district, vic. Voskresenka vill., 55°19'N; 86°48'E, July 2019. A. Polevoi leg.
C. myrtilli	CMKEM_f5.7	f	-	-		RUSSIA, Kemerovo region, Kemerovskiy district, vic. Voskresenka vill., 55°19'N; 86°48'E, July 2019. A. Polevoi leg.
C. myrtilli	CMKEM_f5.8	f	-	-		RUSSIA, Kemerovo region, Kemerovskiy district, vic. Voskresenka vill., 55°19'N; 86°48'E, July 2019. A. Polevoi leg.
C. myrtilli	CMKEM_f5.9	f	-	-		RUSSIA, Kemerovo region, Kemerovskiy district, vic. Voskresenka vill., 55°19'N; 86°48'E, July 2019. A. Polevoi leg.
C. myrtilli	CMKEM_f5.10	f	-	-		RUSSIA, Kemerovo region, Kemerovskiy district, vic. Voskresenka vill., 55°19'N; 86°48'E, July 2019. A. Polevoi leg.
C. myrtilli	CMKEM_f5.11	f	-	-		RUSSIA, Kemerovo region, Kemerovskiy district, vic. Voskresenka vill., 55°19'N; 86°48'E, July 2019. A. Polevoi leg.
C. myrtilli	CMKEM_f5.12	f	-	-		RUSSIA, Kemerovo region, Kemerovskiy district, vic. Voskresenka vill., 55°19'N; 86°48'E, July 2019. A. Polevoi leg.
C. myrtilli	CM6.1	m	-	-		KAZAKHSTAN, Zyryanovsky district, ca. 30 km N of Zyryanovsk city, 50°00'05"N; 84°13'32"E, 11.07.2012, V. Lukhtanov leg
C. myrtilli	CM6.2	f	-	-		KAZAKHSTAN, Zyryanovsky district, ca. 30 km N of Zyryanovsk city, 50°00'05"N; 84°13'32"E, 11.07.2012, V. Lukhtanov leg
C. myrtilli	CM6.3	f	-	-		KAZAKHSTAN, Zyryanovsky district, ca. 30 km N of Zyryanovsk city, 50°00'05"N; 84°13'32"E, 11.07.2012, V. Lukhtanov leg
C. myrtilli	CM6.4	f	-	-		KAZAKHSTAN, Zyryanovsky district, ca. 30 km N of Zyryanovsk city, 50°00'05"N; 84°13'32"E, 11.07.2012, V. Lukhtanov leg
C. myrtilli	CM6.5	f	-	-		KAZAKHSTAN, Zyryanovsky district, ca. 30 km N of Zyryanovsk city, 50°00'05"N; 84°13'32"E, 11.07.2012, V. Lukhtanov leg
C. myrtilli	CM6.6	f	-	-		KAZAKHSTAN, Zyryanovsky district, ca. 30 km N of Zyryanovsk city, 50°00'05"N; 84°13'32"E, 11.07.2012, V. Lukhtanov leg
C. myrtilli	CM6.7	f	-	-		KAZAKHSTAN, Zyryanovsky district, ca. 30 km N of Zyryanovsk city, 50°00'05"N; 84°13'32"E, 11.07.2012, V. Lukhtanov leg
C. myrtilli	CM6.8	f	-	-		KAZAKHSTAN, Zyryanovsky district, ca. 30 km N of Zyryanovsk city, 50°00'05"N; 84°13'32"E, 11.07.2012, V. Lukhtanov leg
C. myrtilli	CM6.9	f	-	-		KAZAKHSTAN, Zyryanovsky district, ca. 30 km N of Zyryanovsk city, 50°00'05"N; 84°13'32"E, 11.07.2012, V. Lukhtanov leg
C. myrtilli	CM6.10	f	+	+		KAZAKHSTAN, Zyryanovsky district, ca. 30 km N of Zyryanovsk city, 50°00'05"N; 84°13'32"E, 11.07.2012, V. Lukhtanov leg
C. myrtilli	CM6.11	f	-	-		KAZAKHSTAN, Zyryanovsky district, ca. 30 km N of Zyryanovsk city, 50°00'05"N; 84°13'32"E, 11.07.2012, V. Lukhtanov leg

C. myrtilli	CMM1_f1	f	-	-		CZECH REPUBLIC, Karlovy Vary region, Kleiner Kranichsee Natural Reserve, 50°24'56"N; 12°40'30"E, h = 925 m, 28.07.2020, I. Malinovsky leg.
C. myrtilli	CMM1_f2	f	-	-		CZECH REPUBLIC, Karlovy Vary region, Kleiner Kranichsee Natural Reserve, 50°24'56"N; 12°40'30"E, h = 925 m, 28.07.2020, I. Malinovsky leg.
C. myrtilli	CMM1_f3	f	-	-		CZECH REPUBLIC, Karlovy Vary region, Kleiner Kranichsee Natural Reserve, 50°24'56"N; 12°40'30"E, h = 925 m, 28.07.2020, I. Malinovsky leg.
C. myrtilli	CMM1_f4	f	-	-		CZECH REPUBLIC, Karlovy Vary region, Kleiner Kranichsee Natural Reserve, 50°24'56"N; 12°40'30"E, h = 925 m, 28.07.2020, I. Malinovsky leg.
C. myrtilli	CMM1_f5	f	-	-		CZECH REPUBLIC, Karlovy Vary region, Kleiner Kranichsee Natural Reserve, 50°24'56"N; 12°40'30"E, h = 925 m, 28.07.2020, I. Malinovsky leg.
C. myrtilli	CMM1_f6	f	-	-		CZECH REPUBLIC, Karlovy Vary region, Kleiner Kranichsee Natural Reserve, 50°24'56"N; 12°40'30"E, h = 925 m, 28.07.2020, I. Malinovsky leg.
C. myrtilli	CMM1_f7	f	-	-		CZECH REPUBLIC, Karlovy Vary region, Kleiner Kranichsee Natural Reserve, 50°24'56"N; 12°40'30"E, h = 925 m, 28.07.2020, I. Malinovsky leg.
C. myrtilli	CMM1_f8	f	-	-		CZECH REPUBLIC, Karlovy Vary region, Kleiner Kranichsee Natural Reserve, 50°24'56"N; 12°40'30"E, h = 925 m, 28.07.2020, I. Malinovsky leg.
C. myrtilli	CMM1_f9	f	-	-		CZECH REPUBLIC, Karlovy Vary region, Kleiner Kranichsee Natural Reserve, 50°24'56"N; 12°40'30"E, h = 925 m, 28.07.2020, I. Malinovsky leg.
C. myrtilli	CMM1_f10	f	-	-		CZECH REPUBLIC, Karlovy Vary region, Kleiner Kranichsee Natural Reserve, 50°24'56"N; 12°40'30"E, h = 925 m, 28.07.2020, I. Malinovsky leg.
C. myrtilli	CMM1_f11	f	-	-		CZECH REPUBLIC, Karlovy Vary region, Kleiner Kranichsee Natural Reserve, 50°24'56"N; 12°40'30"E, h = 925 m, 28.07.2020, I. Malinovsky leg.
C. myrtilli	CMM1_f12	f	-	-		CZECH REPUBLIC, Karlovy Vary region, Kleiner Kranichsee Natural Reserve, 50°24'56"N; 12°40'30"E, h = 925 m, 28.07.2020, I. Malinovsky leg.
C. myrtilli	CMM2_f1	f	-	-		CZECH REPUBLIC, Karlovy Vary region, Kleiner Kranichsee Natural Reserve, 50°23'33"N; 12°37'48"E, h = 915 m, 29.07.2020, I. Malinovsky leg.
C. myrtilli	CMM2_f2	f	-	-		CZECH REPUBLIC, Karlovy Vary region, Kleiner Kranichsee Natural Reserve, 50°23'33"N; 12°37'48"E, h = 915 m, 29.07.2020, I. Malinovsky leg.
C. myrtilli	CMM2_f3	f	-	-		CZECH REPUBLIC, Karlovy Vary region, Kleiner Kranichsee Natural Reserve, 50°23'33"N; 12°37'48"E, h = 915 m, 29.07.2020, I. Malinovsky leg.
C. myrtilli	CMM2_f4	f	-	-		CZECH REPUBLIC, Karlovy Vary region, Kleiner Kranichsee Natural Reserve, 50°23'33"N; 12°37'48"E, h = 915 m, 29.07.2020, I. Malinovsky leg.
C. myrtilli	CMM2_f5	f	-	-		CZECH REPUBLIC, Karlovy Vary region, Kleiner Kranichsee Natural Reserve, 50°23'33"N; 12°37'48"E, h = 915 m, 29.07.2020, I. Malinovsky leg.
C. myrtilli	CMM2_f6	f	-	-		CZECH REPUBLIC, Karlovy Vary region, Kleiner Kranichsee Natural Reserve, 50°23'33"N; 12°37'48"E, h = 915 m, 29.07.2020, I. Malinovsky leg.
C. myrtilli	CMM2_f7	f	-	-		CZECH REPUBLIC, Karlovy Vary region, Kleiner Kranichsee Natural Reserve, 50°23'33"N; 12°37'48"E, h = 915 m, 29.07.2020, I. Malinovsky leg.

C. myrtilli	CMM2_f8	f	-	-		CZECH REPUBLIC, Karlovy Vary region, Kleiner Kranichsee Natural Reserve, 50°23'33"N; 12°37'48"E, h = 915 m, 29.07.2020, I. Malinovsky leg.
C. myrtilli	CMM2_f9	f	-	-		CZECH REPUBLIC, Karlovy Vary region, Kleiner Kranichsee Natural Reserve, 50°23'33"N; 12°37'48"E, h = 915 m, 29.07.2020, I. Malinovsky leg.
C. myrtilli	CMM2_f10	f	-	-		CZECH REPUBLIC, Karlovy Vary region, Kleiner Kranichsee Natural Reserve, 50°23'33"N; 12°37'48"E, h = 915 m, 29.07.2020, I. Malinovsky leg.
C. myrtilli	CMM2_f11	f	-	-		CZECH REPUBLIC, Karlovy Vary region, Kleiner Kranichsee Natural Reserve, 50°23'33"N; 12°37'48"E, h = 915 m, 29.07.2020, I. Malinovsky leg.
C. myrtilli	CMM2_f12	f	-	-		CZECH REPUBLIC, Karlovy Vary region, Kleiner Kranichsee Natural Reserve, 50°23'33"N; 12°37'48"E, h = 915 m, 29.07.2020, I. Malinovsky leg.
C. myrtilli	CMRILA_f1	f	n\a	-		BULGARIA, Kyustendil province, Rila, Stara planina Mts., 42°06'N; 23°33'E, 17.08.2020, I. Gjonov leg.
C. myrtilli	CMRILA_f2	f	n\a	-		BULGARIA, Kyustendil province, Rila, Stara planina Mts., 42°06'N; 23°33'E, 17.08.2020, I. Gjonov leg.
C. myrtilli	CMRILA_f3	f	n\a	-		BULGARIA, Kyustendil province, Rila, Stara planina Mts., 42°06'N; 23°33'E, 17.08.2020, I. Gjonov leg.
C. myrtilli	CMRILA_f4	f	n\a	-		BULGARIA, Kyustendil province, Rila, Stara planina Mts., 42°06'N; 23°33'E, 17.08.2020, I. Gjonov leg.
